# Synthesis, Characterization, and Catalytic Activity of Gold Complexes Bearing Bicyclic Silicon and Germanium Anionic Ligands

**DOI:** 10.1021/acs.organomet.5c00299

**Published:** 2025-10-03

**Authors:** Pamela Adienes Benzan Lantigua, Martin Lutz, Marc-Etienne Moret

**Affiliations:** † Organic Chemistry and Catalysis, Institute for Sustainable and Circular Chemistry, Faculty of Science, Utrecht University, Universiteitsweg 99, 3584 CG Utrecht, The Netherlands; ‡ Structural Biochemistry, Bijvoet Centre for Biomolecular Research, Faculty of Science, Utrecht University, Universiteitsweg 99, 3584 CG Utrecht, The Netherlands

## Abstract

Silanides ([R_3_Si^–^]) and germanides ([R_3_Ge^–^]) are attracting attention as donor ligands for transition metals, but their use as supporting ligands in catalysis is limited, often due to their high reactivity. In this study, we report the preparation and characterization of gold­(I) complexes bearing the anionic silanide and germanide ligands tmimSi^–^ and tmimGe^–^ (tmimH_3_ = tris­(3-methylindol-2-yl)­methane), which are stabilized by a bicyclic cage structure. Both the silanide and germanide complexes catalyze the hydroamination of 1-ethynyl-4-fluorobenzene with aniline. The germanide complexes are more efficient than their silanide analogues, likely due to their more electrophilic character. These results show that both ligand types can be used as supporting ligands in catalysis, which warrants further investigations.

## Graphical abstract

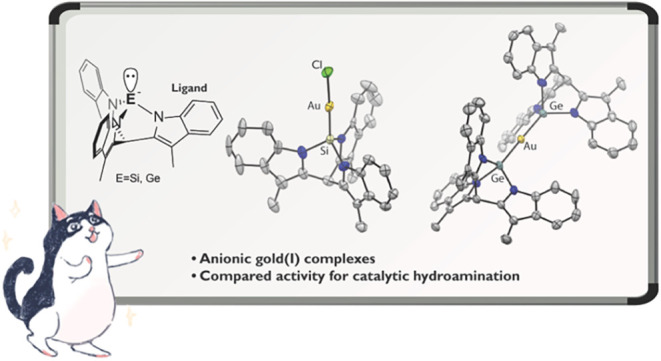

## Introduction

Gold^1^ had long been considered inert and thus of limited synthetic utility, but recent decades have seen widespread applications of gold complexes in homogeneous catalysis to build molecular complexity.^2–38^ Many gold-catalyzed reactions are based on the carbophilic Lewis acidity of gold, which is typically used to activate alkynes, alkenes, allenes, etc. toward a nucleophilic attack as the initial step.^9,22,23,25,39–45^ While simple salts like AuCl can be effective and have remained successful as the field progressed, linear Au­(I) complexes of organic ligands such as phosphine^44,46–52^ and heterocyclic carbenes^32,33,44,53–59^ allow fine-tuning of both activity and selectivity.^8,60–63^ In addition, ligands stabilize the active catalyst against decomposition (e.g., to metal particles).^8,64^ To unlock catalytic activity, it is often necessary to abstract one ligand (typically Cl^–^) from the gold precatalyst to generate a reactive cationic center.^65,66^

Low-valent silicon­(II) compounds such as (base-stabilized) silylenes are emerging as strongly donating supporting ligands for transition metal centers in homogeneous catalysis.^67–71^ They often surpass the vastly used phosphine and carbene ligands in activity and selectivity because of their exceptionally strong σ-donor character^72,73^ and high trans-effect.^74^ Germanium­(II) compounds are generally less reducing, but also weaker σ-donors than the Si­(II) analogues, and are therefore less broadly explored as ligands.^75^

There are some reported examples of group 11 complexes incorporating ligands with a low-valent silicon or germanium atom, but their coordination chemistry with Au remains underexplored.^76–81^ One of the few examples is the study by Khan et al. reporting the synthesis of a series of Au cations supported by silylene and germylene and their uses as catalysts for the activation of terminal alkynes in glycoside synthesis (Figure 1a).^77^ Iwamoto et al. also reported the synthesis, structure, and properties of gold complexes ligated by cyclic alkylsilylenes (Figure 1b).^82^

**Figure 1:**
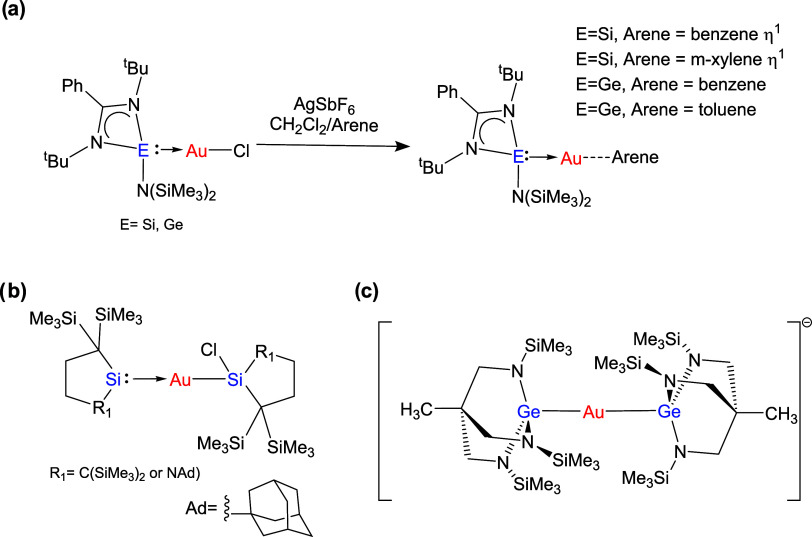
Examples of gold complexes incorporating (base-stabilized) silylene (a, b), germylene (a), or germanide (c) ligands.

Anionic Si­(II) (silanides) and Ge­(II) (germanides) compounds are generally donors that are even stronger than their neutral analogues. They indeed form strongly covalent Au–Si and Au–Ge bonds, but these bonds are often reactive, precluding the use of Si and Ge anions as supporting ligands for catalysis. However, these anionic ligands could be attractive to generate neutral active complexes that would require no halide abstraction step or, more generally, to stabilize low-coordinate intermediates during the catalytic cycle.^9,53,83,84^ Interestingly, Gade et al. showed that a thermally stable triamidogermanate could be used to form a stable digermaaurate salt (Figure 1c).^85^

We have recently shown that tris­(indolyl) scaffolds afford stabilized anionic Si and Ge centers capable of reversibly coordinating to various transition metals.^75,86^ Hypothesizing that such anionic ligands may (transiently) support overall neutral, Lewis-acidic Au­(I) species in a catalytic cycle, we set out to investigate the gold coordination chemistry of the silicon cage tmimSi^–^K^+^[18-crown-6] (tmimH_3_ = tris­(3-methylindol-2-yl)­methane) and the germanium analogue tmimGe^–^K^+^. We show that both monoligated and bisligated species are accessible. Using alkyne hydroamination as a test reaction, we show that both ligands can support electrophilic gold catalysis, the Ge analogues affording better activity, presumably because of their higher electrophilicity.

## Results and Discussion

### Complex Synthesis

The silanide ligand tmimSi^–^K^+^[18-crown-6] (**1**) and the analogue germanide tmimGe^–^K^+^ (**1′**) were synthesized using literature procedures.^75,86^ The reaction of AuCl­(SMe_2_) with one equivalent of silanide **1** or germanide **1′** afforded the anionic complexes **2** and **5**, respectively (Scheme 1), which were characterized by NMR spectroscopy (^1^H, ^13^C, and ^29^Si if applicable), electrospray ionization mass spectrometry (ESI-MS), and X-ray crystallography. The ^1^H NMR spectra show a single set of four signals in the aromatic region, indicating that the *C*_3_ symmetry of the cage ligand is maintained in both complexes. Moreover, both complexes display a single R_3_C*H* resonance (5.93 ppm for **2** and 6.10 ppm for **5**). The ^29^Si NMR signal of **2** at −17.4 ppm is significantly deshielded as compared with the free silanide ligand (−48.1 ppm). ESI-MS analyses are consistent with a monomeric anionic complex in both cases.

**Scheme 1:**
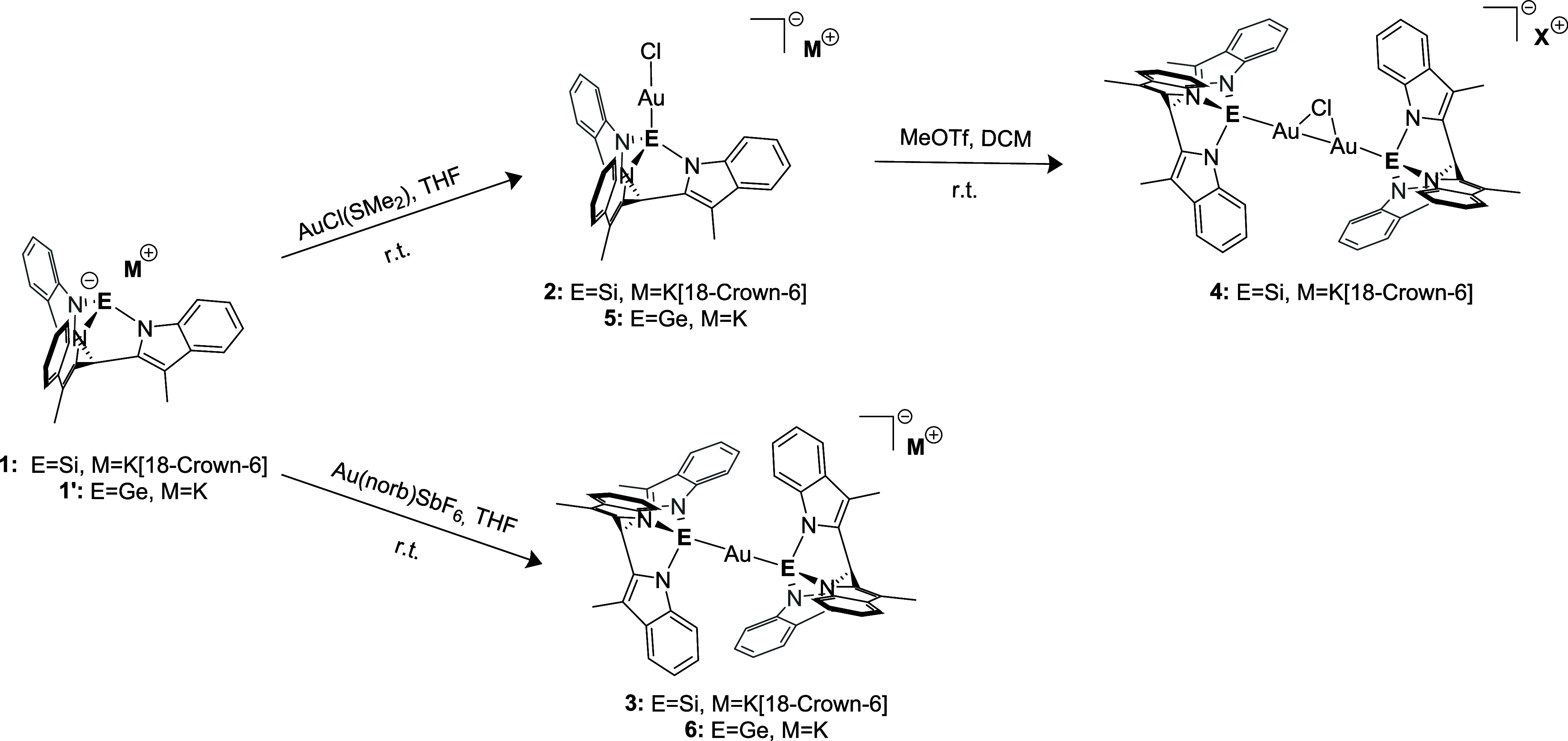
Gold Complexation of Silanide **1** and Germanide **1′**

An X-ray crystal structure of **2** shows two independent molecules in the asymmetric unit and a weak interaction between the ions (see the Supporting Information (SI) Section 4.1). One of the molecular anions interacts with both [18-crown-6]­K^+^ counterions via π-complexation of the indole rings, while the other one is free of such interactions. The structure displays a two-coordinate Au­(I) complex with one coordination site occupied by the silanide ligand and the other coordination site occupied by the chloride ligand (Figure 2). Si–Au–Cl angles of 177.55(10)/172.57(11)° indicate a linear geometry, as is common for Au­(I) complexes. The Si–Au distances of 2.230(3)/2.242(2) Å are consistent with most of the previously observed gold­(I) silyl complexes with a linear Si–Au–Cl arrangement.^79^

**Figure 2:**
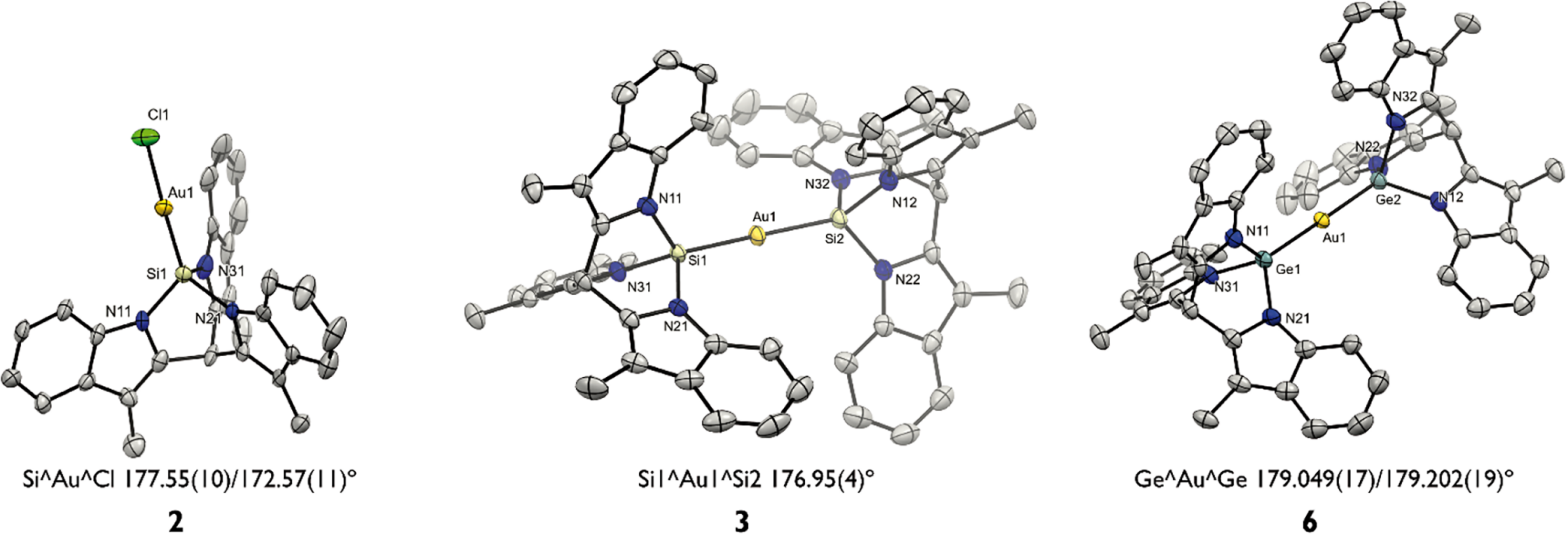
Molecular structures of compounds **2**, **3**, and **6**. Displacement ellipsoids are drawn at the 50% probability level. Counteranions, solvents, and hydrogen atoms were omitted for clarity.

The complex **5** was crystallized by vapor diffusion of *n*-hexane in THF. The crystal structure contains three independent molecules in the asymmetric unit. The THF molecules are severely disordered. The structure shows a linear geometry at the Au­(I) center with a Ge–Au–Cl angle (178.32(7), 175.43(9), 178.62(7)°) consistent with what was observed in other reported complexes with Ge­(II) as a ligand. For both **2** and **5**, the N–E–N angles (E = Si or Ge) increase upon complexation, presumably due to an increase of the p-character of the lone pair and a consequent decrease in the p-character of the E–N bonding orbital (from Σ = 272.5(8)° in the free Si ligand^86^ to Σ = 288.5(7)–288.6(5)° in **2**; and from 263.9(6)° in the free Ge ligand^75^ to Σ = 278.5(4)–279.4(4)° in **5**).

The bisligated complexes **3** and **6**, each of which contains one gold center and two ligands, were synthesized using [Au­(norb)_3_]­SbF_6_ ^87^ (norb = norbornene) as the metal precursor (Scheme 1). Their ^1^H NMR spectra each show a single set of signals in the aromatic region, indicating that the 3-fold symmetry of the cage ligand is preserved in both complexes. The ^29^Si NMR signal of **3** at 40.1 ppm is significantly deshielded compared to the free silanide ligand (−48.1 ppm) and the monoligated complex **2** (−17.4 ppm). The X-ray crystal structures of **3** and **6** (Figure 2) both show a two-coordinate Au­(I) complex with both coordination sites occupied by silanide or germanide ligands, respectively. Weak interactions are present between the K^+^[18-crown-6] cation and the ligands. The linearity is reflected in the E–Au–E angle (E = Si, 176.95(4)°; E = Ge 179.049(17)/179.202(19)°). The E–Au distances are longer than the distance in the analogous monoligated complexes, suggesting weaker metal–ligand interactions (from 2.230(3) Å in **2** to 2.3402(12)–2.3425(12) Å in **3**; from 2.3039(7)–2.3096(6) Å in **5** to 2.3909(4)–2.3997(5) Å in **6**). The Ge–Au bond in **6** is shorter compared to the gold bis-germanide complex (Figure 1c) reported by Gade et al.^85^ ([2.423(2) Å]) and even shorter than the values determined by Schmidbaur et al. for (Ph_3_P)­AuGeCl_3_ ([2.406(1) Å]) and (Ph_3_P)_3_AuGeCl_3_ ([2.536(1) Å]).^88^

In gold catalysis, it is often necessary to abstract one ligand from a gold species of the type LAuX to induce sufficient reactivity. Silver salts are often used, but they might not be innocent during catalysis; moreover, incomplete halide abstraction can occur.^66,89^ Several attempts to abstract the halide ligand in **2** to isolate a chloride-free species using different Ag salts, Na salts, and GaCl_3_ were unsuccessful. Only the reaction of the complex **2** with MeOTf (a strong electrophilic methylating agent) somewhat unexpectedly afforded the bimetallic complex **4**. The combination of ^1^H NMR and ^29^Si NMR spectroscopy, in addition to the obtained X-ray crystal structure (see the SI), even though of low quality, supports a bimetallic structure in which two [tmimSiAu] fragments are bridged by a single chloride ligand. This structure is also supported by the detection of MeCl formed during the synthesis of the complex (see the SI). The ESI-MS analysis of the isolated compound showed the nominal *m*/*z* of triflate (149) and of the complex **2** (660 for C_28_H_22_AuClN_3_Si^–^), indicating that the complex fragments under the measurement conditions.

Examples of this type of halide-bridged complexes are reported both with chelating^90–92^ and nonchelating ligands.^93^ The Au–Au distances that are considered in the range for an aurophilic interaction have been defined by Schmidbaur to be 2.5–3.5.^94^ Moreover, in complexes where multiple gold atoms coordinate around a single central atom, aurophilic interactions are also indicated by acute Au–X–Au angles (<90°).^89^ In the crystal structure of **4**, the Au–Cl–Au angle is 80.5(2)° and the Au–Au distance is 3.082(2) Å, both of which suggest the presence of an aurophilic interaction.

NBO analyses of the Si and Ge monoligated and bisligated anionic complexes **2, 3, 5**, and **6** showed that the linear coordination at Au is best described as a 3c/4e-bonding situation (ω-type bond) consisting of the resonance structures: L_1_–A_u_:L_2_ ↔ L_1_:A_u_–L_2_. It is characterized by a high occupancy of the σ_Au–L1_ bonding orbital, a relatively low occupancy of the LP_L2_ lone pair, and a high LP_L_2__ → σ_Au–L_1__^*^ second-order perturbation energy (see the SI). For all complexes, the σ_Au–L1_ orbital is strongly polarized toward E (Si or Ge), even though Au is more electronegative than Si or Ge, illustrating the stabilizing effect of the tmim cage on the tetrel lone pair. Moreover, the LP_L_2__ → σ_Au–L_1__^*^ second-order perturbation energy is much higher for the bisligated species **3** and **6** compared to the monoligated ones **2** and **5**. This suggests that the elongation of the Au–L1 bond observed in the crystal structures of **3** and **6** compared to those of **2** and **5** originates from the competition of both tetrel lone pairs for the accepting Au-centered orbital.

### Hydroamination Catalysis

Alkyne hydroamination represents a simple one-step conversion of inexpensive and readily available substrates and is 100% atom-efficient. Moreover, this reaction has a considerable precedent in literature with several benchmark gold complexes.^40,51,52,84,95–97^ For these reasons, this reaction was selected to probe the catalytic properties of the new Au complexes. Since the tested ligands have a negative charge, we expect a neutral active species (LAu) instead of the usually positively charged one (LAu^+^). We tested the [**L**AuCl]^−^ **M** ^+^ (**2, 5**), [**L**_2_Au]^−^ **M** ^+^ (**3, 6**), and [(**L**Au)_2_Cl]^−^ **M** ^+^ (**4**) complexes in the hydroamination reaction (Scheme 2, top). The formation of imine **A** was monitored in the reaction of aniline with 1-ethynyl-4-fluorobenzene (Scheme 2) using ^19^F NMR spectroscopy (see the SI for details).

**Scheme 2:**
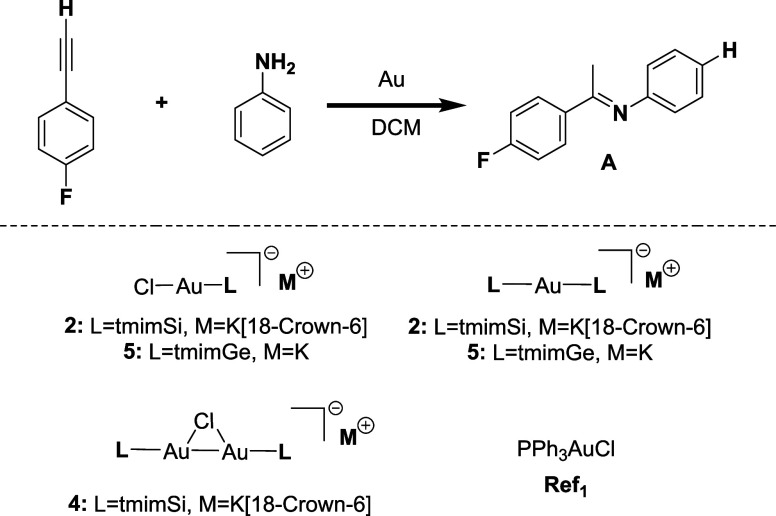
Hydroamination Reaction

The reactions were carried out at room temperature or at 80 °C using AgOTf as a halide abstraction agent (HAA) when indicated. The chosen catalytic loading of 1 mol % is competitive compared to other known systems which often need loadings of 5 mol %.^22,25,39,52^ For comparison, the common gold precatalyst PPh_3_AuCl (**ref**_**1**_) combined with AgOTf affords a yield of 50% at 80 °C in DCM. Since silver salts used for halide abstraction can also act as Lewis acids for catalysis,^52^ a control experiment was performed using only AgOTf (Table 1, entry 10). Under our reaction conditions, AgOTf affords a low yield (20%), after which the reaction stops.

**Table 1:** Hydroamination Reaction

entry	complex	HAA	solvent	temperature	NMR yield (4 h)	NMR yield (22 h)^c^
1	**2** (1% mol)	-	DCM	r.t.	0%	1%
2	**2** (1% mol)	-	DCM	80 °C	2%	2%
3	**2** (1% mol)	AgOTf	DCM	80 °C	31%	41%^b^
4	**3** (1% mol)	-	DCM	80 °C	0%	0%
5	**4** (1% mol)	-	DCM	80 °C	45^**%**^	65%^b^
6	**5** (1% mol)	AgOTf	MeCN-*d*_3_	80 °C	68% (3 h)^a^ ^,^ ^b^	-
7	**6** (1% mol)	-	MeCN-*d*_3_	80 °C	39%	62%
8	**5** (1% mol)	AgOTf	DCM-*d*_2_	80 °C	87% (2 h)^a^	-
9	**ref**_**1**_ (1% mol)	AgOTf	DCM	80 °C	50% (3 h)^a^	-
10	**-**	AgOTf (1% mol)	DCM-*d*_2_	80 °C	20%^b^	-

a The conversion of the starting material stops after the reported reaction time.

b Some other minor products are observed by ^19^F NMR.

c For all experiments, the products were also detected using GC-MS analysis of the extracted organic fractions as the control.

In the absence of an HAA, the complex **2** showed very low activity at r.t. and at 80 °C (Table 1, entries 1 and 2). When the reaction was performed in the presence of AgOTf at 80 °C, the conversion increased to 41% after 22 h (Table 1, entry 3). The bisligated complex **3** afforded no conversion (Table 1, entry 4), suggesting that the silanide ligands are too strongly bound to the metal center to dissociate under these conditions. Some cases are reported in the literature in which the formation of a bisligated complex leads to the deactivation of the catalyst.^79^ The Cl-bridged dinuclear complex **4** showed a higher conversion (65% after 22 h, Table 1, entry 5) compared to **2**, supporting the idea that it can act as a source of the active tmimSi–Au fragment. Since the Au–Cl–Au bridge can be cleaved by coordinating substrates, they can be good catalysts, even though they are usually found less active than the mononuclear LAuCl complexes.^66^

In general, the germanium complexes were found to be more efficient than the corresponding silanide complexes (Table 1). Initially, due to the lower solubility of the Ge complexes in DCM, the reactions using the Ge ligands were performed in MeCN-*d*_3_; however, to have a more direct comparison with the silanides’ results, the reaction of the monoligated complex **5** was also performed in DCM-*d*_2_, obtaining an even better performance. In fact, for the monoligated complex **5** bearing the germanium ligand, the conversion was 87% after 2 h (Table 1, entry 8), while for the corresponding silicon one (complex **2**, entry 2), the conversion was much slower, reaching only 31% after 4 h. Interestingly, the bisligated germanide complex **6** (entry 7) displayed appreciable activity (up to 62% yield) without an HAA, suggesting that one Au–Ge bond can be cleaved under the reaction conditions.

Mechanistically, gold-catalyzed hydroamination generally involves alkyne π-coordination to a Lewis-acidic gold center followed by nucleophilic attack to generate an aminovinyl intermediate and protodeauration to release the product (Scheme 3). Ligand effects on gold-catalyzed hydroamination reactions depend on which step is rate-limiting; protodeauration^84^ is accelerated by electron-releasing ligands, while nucleophilic attack is favored by electron-withdrawing ligands. Since Au complexes of the less electrodonating germanide ligand^75,86^ are more active than the silanide analogues, nucleophilic attack is likely rate-limiting here.

**Scheme 3:**
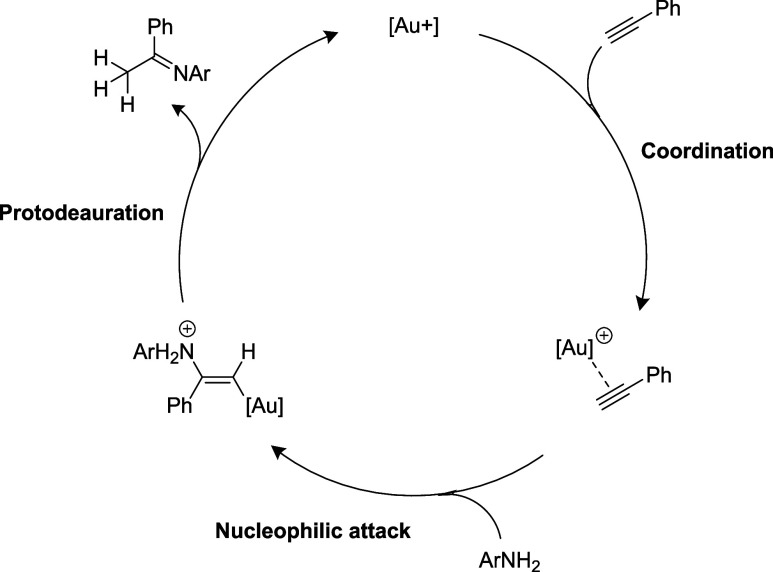
Schematic Hydroamination Mechanism

In support of this interpretation, the phenylacetylene complexes with neutral (tmim)­SiAu and (tmim)­GeAu were investigated computationally (see the SI for details). In both cases, the LUMO^52^ is mostly an alkyne π* orbital with a bonding component to Au and is most likely the orbital involved in nucleophilic attack. The highest occupied molecular orbital–lowest unoccupied molecular orbital (HOMO–LUMO) gap for Ge is significantly smaller (45.4 kcal/mol) compared to Si (55.3 kcal/mol), which is consistent with a higher activity of the Ge compound (see the SI). In addition, a stronger donation from the C–C π bond toward the Au–Ge antibonding orbital was observed (41.0 kcal/mol) compared to the Au–Si one (30.2 kcal/mol), making the alkyne more electrophilic. It should be noted that other factors may contribute to the higher activity of the germanide complexes. Since germanium­(II) is less reducing than silicon­(II), it may provide better stabilization against Au(0) precipitation. In addition, the observed activity of the bisligated germanide complex **6** indicates that the putative formation of this species after partial gold(0) precipitation would not completely quench the catalytic activity.

## Conclusions

In conclusion, it was demonstrated that the silanide ligand tmimSi^–^K^+^[18-crown-6] (**1**) and the analogue germanide tmimGe^–^K^+^ (**1′**) can form stable monoligated and bisligated Au­(I) complexes. Moreover, partial halide abstraction from the monoligated complex (tmim)­SiAuCl (**2**) by MeOTf afforded a dinuclear, chloride-bridged complex. A catalytic application of the new gold complexes was demonstrated with the intermolecular hydroamination of 1-ethynyl-4-fluorobenzene with aniline. The germanide complexes were found more efficient than their silanide analogues, probably due to their more electrophilic nature. These findings demonstrate the ability of stabilized silanide and germanide ligands to support metal-centered catalysis, warranting further investigations in the future.

## Supplementary Material





## Notes

Experimental procedures, NMR spectra, and supplementary experimental and computational data (PDF)
Cartesian coordinates of optimized structures (XYZ)
