# Supplementary material for: Synthesis, Characterization, and Catalytic Activity of Gold Complexes Bearing Bicyclic Silicon and Germanium Anionic Ligands
Source: Organometallics. 2025 Oct 3;44(19):2287–94. doi: 10.1021/acs.organomet.5c00299 (PMC12522683; doi:10.1021/acs.organomet.5c00299)
Supplement: Supplementary file 1 [file om5c00299_si_001.pdf]

Supporting Information

Synthesis, Characterization, And Catalytic Activity Of Gold Complexes Bearing Bicyclic Silicon And Germanium Anionic Ligands

Pamela Adienes Benzan Lantigua[a], Martin Lutz[b], Marc-Etienne Moret[a] *

[a] Organic Chemistry and Catalysis, Institute for Sustainable and Circular Chemistry, Faculty of Science, Utrecht University, Universiteitsweg 99, 3584 CG, Utrecht (The Netherlands), E-mail: M.Moret@uu.nl

[b] Structural Biochemistry, Bijvoet Centre for Biomolecular Research, Faculty of Science, Utrecht University, Universiteitsweg 99, 3584 CG, Utrecht (The Netherlands)

1. Experimental section

1.1. General information

All reactions involving air-sensitive compounds were conducted under an N_2 atmosphere by using standard glovebox or Schlenk techniques. Acetonitrile, n-hexane and Et_2O were dried with an MBRAUN MB SPS-79 system. THF was distilled from benzophenone/Na. All solvents were degassed by bubbling with N_2 and stored over molecular sieves in a glovebox. Deuterated acetonitrile, THF, and DCM were degassed by four freeze-pump-thaw cycles and stored over molecular sieves in a glovebox. Skatole, KH 30 w% in mineral oil, 2,6-diisopropylaniline, $HSiCl_3$, KOtBu (≥97%), norbonylene, aniline, 1-Ethynyl-4-fluorobenzene, MeOTf and NaOTF were purchased from Sigma-Aldrich. Triethyl orthoformate, glyoxal 40 w%, TMSCl, paraformaldehyde, 18-crown-6, AgOTf and $AgSbF_6$ were purchased from Acros. $AuClS(CH_3)_2$ was purchased from ABCR. All chemicals used in this work except the ligands and the complexes were commercial products of the highest available purity and were further purified by the standard methods, if necessary. The ligand and the precursors were prepared according to the reported procedure[1–4].

1.2. Physical Methods

All NMR measurements were performed on a Varian VNMRS400 or Varian MRF400 spectrometer. The chemical shifts are reported relative to TMS with the residual solvent signal as internal standard. All NMR experiments involving air-sensitive compounds were conducted in J-Young NMR tubes under an N_2 atmosphere. ESI-MS measurements were performed on a Waters LCT Premier XE KE317 spectrometer.

2. Synthesis and Characterization

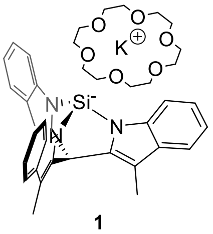

1

Synthesis of $[(tmim)Si]^-[(18\text{-crown-}6)K]^+$ (1). The synthesis was performed as reported by Benzan Lantigua et al. [5]
^{1}H-NMR (400 MHz, CD_3CN) δ 7.73 (dt, *J*= 8.1, 1.0 Hz, 3H, indole-C*H*^{7}), 7.32 (dt, *J* = 7.8, 1.0 Hz, 3H, indole-C*H*^{4}), 6.97 (ddd, *J* = 8.2, 7.0, 1.3 Hz, 3H, indole-C*H*^{5}), 6.87 (ddd, *J* = 7.9, 7.0, 1.1 Hz, 3H, indole-C*H*^{6}), 5.95 (s, 1H, Indole-C*H*$_{linker}$), 3.56 (s, [18-crown-6]), 2.42 (s, 9H, Indole-C*H$_3$*). **^{29}Si{^{1}H}-NMR** (79 MHz, CD_3CN) δ -48.1.

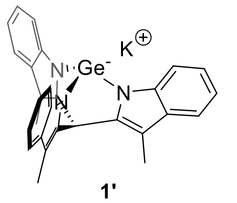

1'

Synthesis of $[(tmim)Si]^-K^+$ (1'). The synthesis was performed as reported by Witteman et al[2].
^{1}H-NMR (400 MHz, CD_3CN) δ = 7.58 (dt, J= 8.1, 0.9, 0.9 Hz) 3H, Indole-C*H*^{7}), 7.34 (dt, *J*= 7.8, 0.9, 0.9 Hz), 3H, Indole-C*H*^{4}), 6.94 (ddd, *J*= 8.1, 6.9, 1.3 Hz, 3H, Indole-C*H*^{6}), 6.85 (ddd, *J*=7.9, 7.0, 1.1 Hz, 3H, Indole-C*H*^{5}), 6.03 (s, 1H, indole-C*H$_{linker}$*), 2.46 ppm (s, 9H, Indole-C*H$_3$*).

Synthesis of $tmimSiAuCl^-K^+$[18-crown-6]. (2) A suspension of $tmimSi^-K^+$[18-crown-6] (94.1 mg, 0.129 mmol) in THF (4 mL) was added to a solution of $AuClS(CH_3)_2$ (37.7 mg, 0.128 mmol) in THF (3 mL) and the mixture was stirred for 30 minutes. The resulting dark grey suspension was filtered through celite, washed with THF (3 × 1 mL). The filtrate was dried, and the resulting grey/green powder was dissolved in the minimum amount of DCM and precipitated using Et_2O. The supernatant was removed, and the precipitate washed with Et_2O. Drying in vacuo afforded **2** as a green powder (84mg, 0.087 mmol, 68 %). Crystals suitable for X-ray crystallography were grown by vapor diffusion of Et_2O into a concentrated solution of **2** in DCM at r.t.

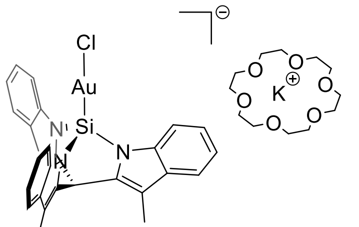

2

^{1}H-NMR (400 MHz, CD_2Cl_2) δ 8.20 (dt, *J* = 8.1, 0.9 Hz, 3H, indole-C*H*^{4}), 7.37 (ddd, *J* = 7.7, 1.3, 0.7 Hz, 3H, indole-C*H*^{7}), 7.07 (ddd, *J* = 8.2, 7.1, 1.3 Hz, 3H, indole-C*H*^{5}), 6.96 (ddd, *J* = 8.0, 7.1, 1.1 Hz, 3H, indole-C*H*^{6}), 5.93 (s, 1H, s, 1H, Indole-C*H*$_{Linker}$), 3.30 (s, 18-crown-6), 2.40 (s, 9H, Indole-C*H$_3$*); **^{13}C{^{1}H}-NMR** (101 MHz, CD_2Cl_2) δ 140.2(s, *C*$_{Indole}$), 139.6(s, *C*$_{Indole}$), 131.1(s, *C*$_{Indole}$), 121.3(s, *C*H$_{Indole}$), 119.0(s, *C*H$_{Indole}$), 118.7(s, *C*H$_{Indole}$), 113.2(s, *C*H$_{Indole}$), 106.3 (s, *C*$_{Indole}$), 70.3 (s, *C*H$_2$,18-crown-6), 32.8 (s, CH$_{Indole}$ linker), 8.7(s, *C*H$_{3\text{-}Indole}$); **^{29}Si{^{1}H}-NMR** (79 MHz, CD_2Cl_2) δ -17.4. **ESI-MS$^-$** ($C_{28}H_{22}AuClN_3Si^-$) m/z calculated 660; found 660.

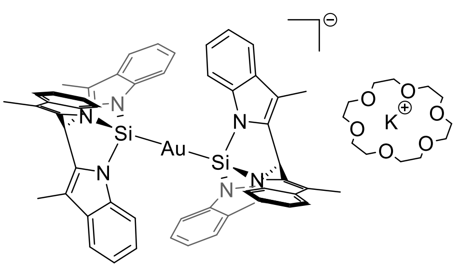

3

Synthesis of $(tmimSi)_2Au^-K^+$[18-crown-6]. (3) A suspension of $tmimSi^-K^+$[18-c-6] (61 mg, 0.083 mmol) in THF (5 mL) was added to a stirring solution of $Au(norb)_3(SbF_6$ (30 mg, 0.042 mmol) in THF (5 mL) and the mixture was stirred for 30 minutes. The resulting dark grey suspension was filtered through celite and washed with THF (3 × 1 mL). The filtrate was dried under vacuum and the resulting light green powder was dissolved in the minimum amount of DCM and precipitated using Et_2O. The supernatant was removed, and the precipitate washed with Et_2O. Drying in vacuo afforded complex **2** as a light green powder (40 mg, 0.029 mmol, 69%). Crystals suitable for X-ray crystallography were grown by vapor diffusion of Et_2O into a concentrated solution of **3** in DCM at room temperature.

^{1}H-NMR (400 MHz, CD_2Cl_2) δ 8.55 – 8.34 (m, 3H, indole-CH^4), 7.48–7.39 (m, 3H, indole-CH^7), 7.11 – 6.90 (m, 6H, indole-CH^6 and indole-CH^5), 6.03 (s, 1H, Indole-CH_{Linker}), 3.58 (s, 18-crown-6), 2.48 (s, 9H, Indole-CH_3); **^{13}C{^{1}H}-NMR** (101 MHz, CD_2Cl_2) δ ^{13}C NMR (101 MHz, CD_2Cl_2) δ 140.6 (s, C_{Indole}), 139.9 (s, C_{Indole}), 131.1 (s, C_{Indole}), 121.7 (s, CH_{Indole}), 119.1 (s, CH_{Indole}) , 118.7 (s, CH_{Indole}), 113.6 (s, CH_{Indole}), 106.8 (s, C_{Indole}), 70.5 (s, CH_2,18-crown-6), 33.0 (s, CH_{Indole} linker), 8.9(s, $CH_{3\text{-}Indole}$); **^{29}Si{^{1}H}-NMR** (79 MHz, CD_2Cl_2) δ 40.1. **ESI- MS$^-$**($C_{56}H_{44}N_6Si_2Au^-$) m/z calculated 1053; found 1053.

Synthesis of $(tmimSi)_2Au_2Cl^-K^+$[18-crown-6]. (4) To a stirring solution of ($tmimSiAuCl^-K^+$[18-crown-6] (51.6 mg, 0.053 mmol) in DCM (3.6 ml) was added MeOTf (5.2 µl, 0.046 mmol) using a microsyringe. The mixture was left stirring over 24h at r.t. The resulting dark purple suspension was filtered through celite and washed with DCM (3 × 1 mL). The filtrate was dried under vacuum and the resulting light purple powder was dissolved in the minimum amount of DCM and precipitated using Et_2O. The supernatant was removed, and the precipitate washed with Et_2O. Drying in vacuo afforded complex **4** as a purple powder (37.2 mg, 0.024 mmol, 92 %). Crystals suitable for X-ray crystallography were grown by vapor diffusion of Et_2O into a concentrated solution of complex **4** in DCM at -40°C.

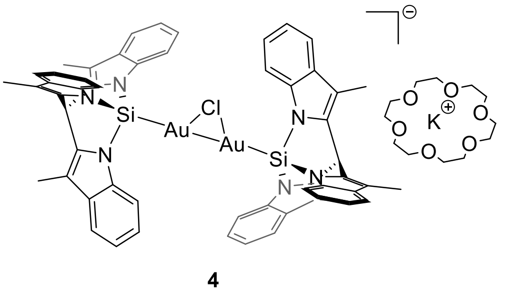

4

^{1}H-NMR δ 8.16 (d, *J* = 7.8 Hz, 3H, indole-CH^4), 7.31 (d, *J* = 7.5 Hz, 3H indole-CH^7), 6.92 – 6.69 (m, 6H, indole-CH^6 and indole-CH^5), 5.93 (s, 1H s, Indole-CH_{Linker}), 3.16 (s, 18-crown-6), 2.38 (s, 9H, Indole-CH_3); **^{13}C{^{1}H}-NMR** (101 MHz, CD_2Cl_2) δ 140.0 (s, C_{Indole}), 139.5 (s, C_{Indole}), 131.2 (s, C_{Indole}) , 121.6 (s, CH_{Indole}), 119.0 (s, CH_{Indole}) , 118.6 (s, CH_{Indole}) , 112.9 (s, CH_{Indole}), 106.8 (s, C_{Indole}), 70.2 (s, CH_2,18-crown-6), 32.8 (s, CH_{Indole} linker), 8.7(s, $CH_{3\text{-}Indole}$); **^{29}Si{^{1}H}-NMR** (79 MHz, CD_2Cl_2) δ -21.8 (br). **ESI- MS$^-$**($C_{56}H_{44}N_6Si_2Au_2Cl^-$) m/z calculated 1285; found 660 and 149.

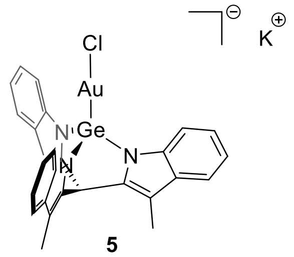

5

Synthesis of $tmimGeAuCl^-K^+$. (5) A solution of of $tmimGe^-K^+$ (59.6 mg, 0.099 mmol, 15% THF) in THF (4 mL) was added to a solution of $AuClS(CH_3)_2$ (24.7mg, mmol) in THF (3 mL) and stirred for 45 minutes. The resulting dark brown suspension was filtered through celite, washed with THF (3 × 1 mL). The filtrate (almost colourless solution with a grey hue) was dried, and the resulting light purple powder was dissolved in the minimum amount of THF and precipitated using *n*-hexane. The supernatant was removed, and the precipitate washed with *n*-hexane. Drying in vacuo afforded **5** as a purple powder (57.1 mg, mmol, 92 %). Crystals suitable for X-ray crystallography were grown by vapor diffusion of *n*-hexane into a concentrated solution of **5** in THF at -40°C.

^{1}H-NMR (400 MHz, CD_3CN) δ 7.83 (dt, *J* = 8.1, 0.9 Hz, 3H, indole-CH^4), 7.40 (dt, *J* = 7.8, 1.0 Hz, 3H, indole-CH^7)), 7.06 (ddd, *J* = 8.1, 7.0, 1.3 Hz, 3H, indole-CH^6), 6.96 (ddd, *J* = 8.0, 7.1, 1.1 Hz, 3H, indole-CH^5), 6.10 (s, 1H, Indole-CH_{Linker}), 2.44 (s, 9H, Indole-CH_3); **^{13}C{^{1}H}-NMR** (101 MHz, CD_3CN) δ 140,5 (s, C_{Indole}), 139.8 (s, C_{Indole}), 131.2 (s, C_{Indole}), 121.7(s, CH_{Indole}), 119.4 (s, CH_{Indole}), 119.3(s, CH_{Indole}), 112.5 (s, CH_{Indole}), 106.3 (s, C_{Indole}), 68.2 (s, -O-CH_2-,THF), 33.1 (s, CH_{Indole} linker), 26.2 (s, CH_2, THF), 8.6 (s, $CH_{3\text{-}Indole}$). **ESI-MS$^-$** ($C_{28}H_{22}AuClGeN_3^-$) m/z calculated 706; found 706.

Synthesis of $(tmimGe)_2Au^-K^+$. (6) The solution of $tmimGe^-K^+$ (86.1 mg, 0.143 mmol, 15% THF) in THF (4 mL) was added to a stirring solution of $Au(norb)_3(SbF_6$ (50.9 mg, 0.071 mmol) in THF (3 mL) and stirred for 45 minutes. The resulting purple suspension was filtered through celite and washed with THF (3 × 1 mL). The filtrate was dried under vacuum and the resulting light grey/purple powder was crystallized in THF/n-hexane. The precipitate was washed with hexane. Drying in vacuo afforded complex **5** as a light white/purple powder (92.5 mg, 0.063 mmol, 88 %, 19% THF). Crystals suitable for X-ray crystallography were grown by vapor diffusion of Et_2O into a concentrated solution of **6** in DCM at room temperature.

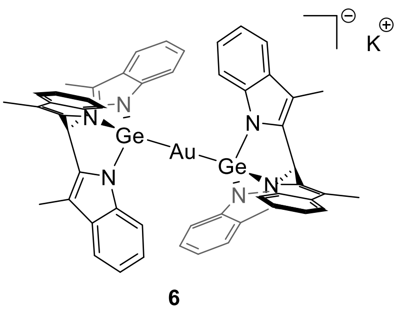

6

^{1}H-NMR (400 MHz, CD_3CN) δ 8.25 (dq, *J* = 8.1, 0.8 Hz, 3H, indole-CH^4), 7.47 (dt, *J* = 7.7, 0.6 Hz, 3H,indole-CH^7), 7.12 (ddd, *J* = 8.2, 7.0, 1.3 Hz, 3H, indole-CH^5), 7.01 (ddd, *J* = 8.0, 7.0, 1.1 Hz, 3H, , indole-CH^6), 6.22 (s, 1H, Indole-CH_{Linker}), 2.52 (s, 9H, Indole-CH_3); **^{13}C-NMR** (101 MHz, CD_3CN) δ 140.6(s, C_{Indole}), 140.1 (s, C_{Indole}), 131.3 (s, C_{Indole}), 121.8 (s, CH_{Indole}), 119.5 (s, CH_{Indole}) , 119.4(s, CH_{Indole}) , 113.3 (s, CH_{Indole}) 106.5 (s, C_{Indole}), 68.6 (s, -O-CH_2-,THF), 33.3 (s, CH_{Indole} linker), 26.2 (s, CH_2, THF), 8.8 (s, $CH_{3\text{-}Indole}$). ESI-MS$^-$($C_{56}H_{44}N_6Ge_2^-$) m/z calculated 1145.2; found 1145.4.

General procedure for the halide abstraction. To a suspension of the tmimSiAuCl$^-$K$^+$[18-crown-6] (20 mg, 1 eq in 2 ml of solvent) a suspension of the halide abstraction agent (AgOTf, $AgSbF_6$, NaOTf, or NaBarF, 1eq in 1 ml of solvent) is added. The mixture is left stirring at r.t. The suspension is filtered through celite, and the filtrate is dried and analysed. If possible, the powder on the filter is extracted and analysed.

General procedure for the hydroamination experiments. In the glovebox, the complex (1% mol) in the appropriate solvent (0.3 ml) was transferred to a J-young tube. Then 1-Ethynyl-4-fluorobenzene (1 eq) and aniline (1eq) were added using a microsyringe, when required the halide abstraction agent AgOTf (1% mol) was added as last. The mixture was stirred and left to react. The reactions were carried out both at r.t and at 80°C. **L**AuCl**M**(**2-5**), **L**$_2$Au**M** (**3-6**), **L**$_2$AuCl**M** (**4**) complexes were used as catalyst. The formation of the product was monitored using ^{1}H-NMR and ^{19}F-NMR spectroscopy. The organic fractions were extracted using *n*-hexane and examined using GC-MS analysis to have a confirmation of the formation of the of the imine A.

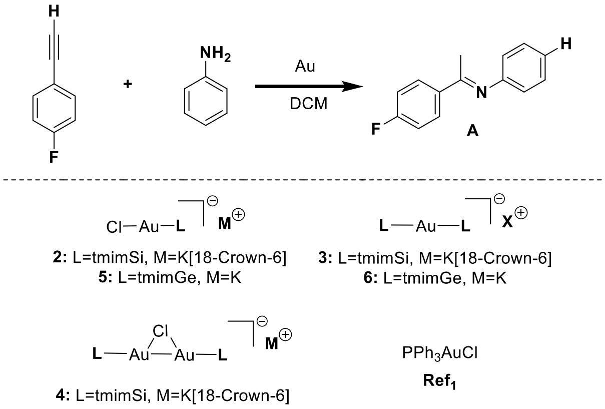

3. Additional experiments

3.1. Reaction of tmimSiAuClk[18-crown-6] with MeOTf: formation of complex 4

The reaction of tmimSiAuClk[18-crown-6] (**2**) with MeOTf was quite clean and showed the formation of complex **4**. The ^{1}H-NMR spectrum shows a single set of signals in the aromatic region in addition to the K^{+}[18-crown-6] counterion, suggesting a retention of the C_3 symmetry. The ^{1}H-NMR spectrum also shows the appearance of a signal at 3.02 ppm that was assigned to MeCl next to the signal at 4.22 ppm that corresponds to MeOTf. Moreover, the ^{19}F-NMR shows and confirms that only half an equivalent of the MeOTf was consumed in the reaction (Figure S1).

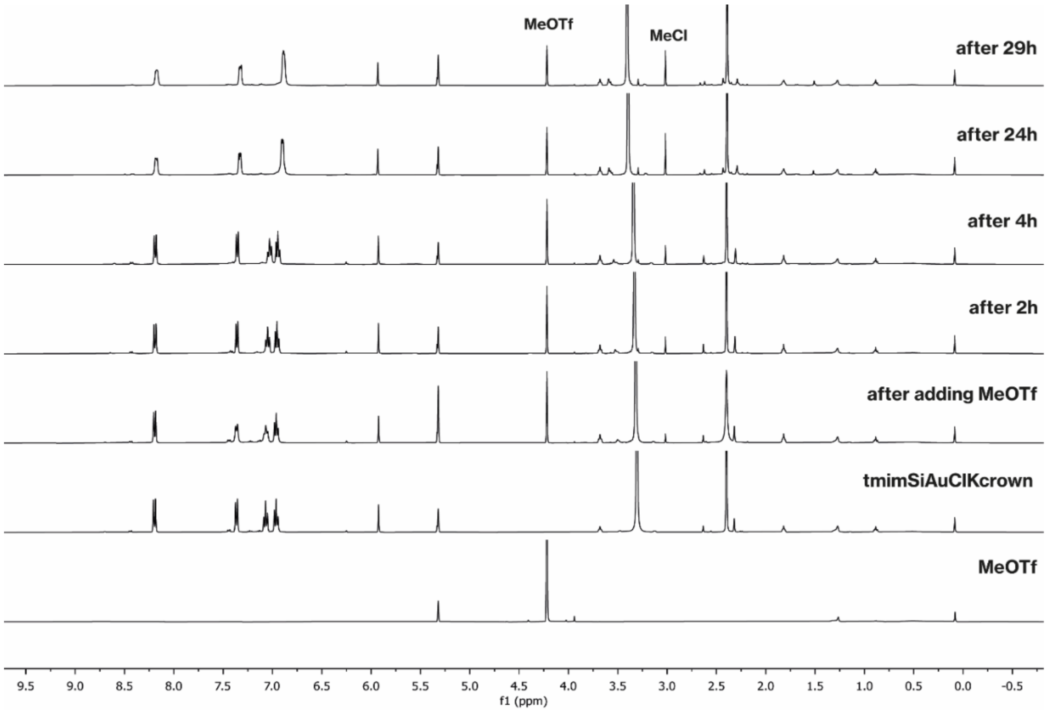

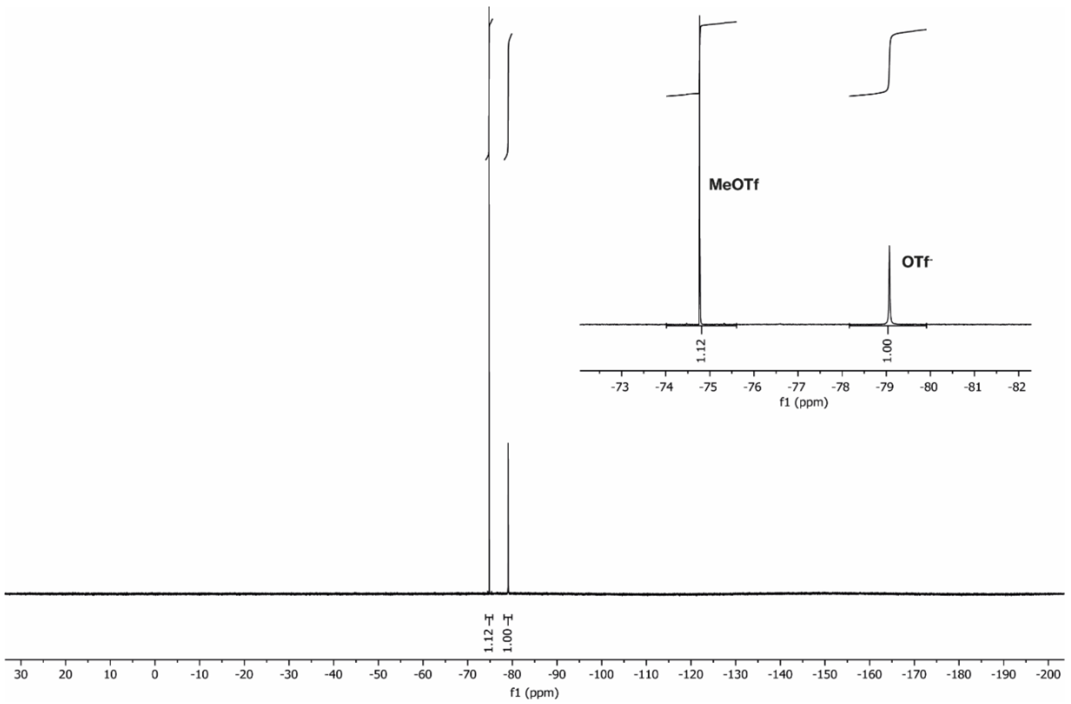

Figure S1. Top: ^{1}H-NMR (400 MHz) spectra of the reaction of compound **2** followed over time in DCM-d_2 at 25°C. For reference: ^{1}H-NMR spectrum of MeOTf, and complex **2** the before and after adding the MeOTf. Bottom: ^{19}F-NMR (376MHz) spectra of the reaction mixture for the formation of **4**

3.2. Halide abstraction reactions

In efforts to remove the chloride ligand, complex **2** was treated with different halide abstraction agents. However, when the reaction was performed in THF with AgX, THF polymerization was observed (see table S1, entry 1 and 7). In presence of other solvents with AgX (Table S1, Entry from 2 to 6) or NaX (table S1 entry from 13 to 18) led to mixtures or the formation of a metallic mirror on the glass walls of the reaction vessel.

Table S1. Halide abstraction reactions and their result

Entry	Reagent	Time	Solvent	Result
**1**	$AgSbF_6$	1h	THF	THF polymerization
**2**	$AgSbF_6$	3h	MeCN	Mixture formation
**3**	$AgSbF_6$	3h	Toluene	Mixture formation
**4**	$AgSbF_6$	3h	DCM	Decomposition (Metallic Mirror formation)
**5**	$AgSbF_6$	Overnight	MeCN	Mixture formation
**6**	$AgSbF_6$	Overnight	Benzene	Decomposition (Metallic Mirror formation)
**7**	AgOTf	1h	THF	THF polymerization
**8**	AgOTf	3h	MeCN	Only complex 2
**9**	AgOTf	3h	Toluene	Mixture formation
**10**	AgOTf	3h	DCM	Decomposition (Metallic Mirror formation)
**11**	AgOTf	Overnight	MeCN	Mixture formation

**12**	AgOTf	Overnight	Benzene	Decomposition (Metallic Mirror formation)
**13**	NaOTf	Overnight	Toluene	Mixture formation
**14**	NaOTf	Overnight	Benzene	Mixture formation
**15**	NaBARF	Overnight	DCM	Mixture formation
**16**	NaBARF	Overnight	Et_2O	Mixture formation
**17**	NaBARF	Overnight	Toluene	Mixture formation
**18**	NaBARF	Overnight	Benzene	Mixture formation

4. X-Ray Crystal structure determinations

CCDC 2476820-2476824 contains the supplementary crystallographic data for this paper. These data can be obtained free of charge from The Cambridge Crystallographic Data Centre via www.ccdc.cam.ac.uk/data_request/cif.

4.1. X-ray crystal structure determination of [(tmim)SiAuCl]$^-$[(18-crown-6)K]$^+$ (2)

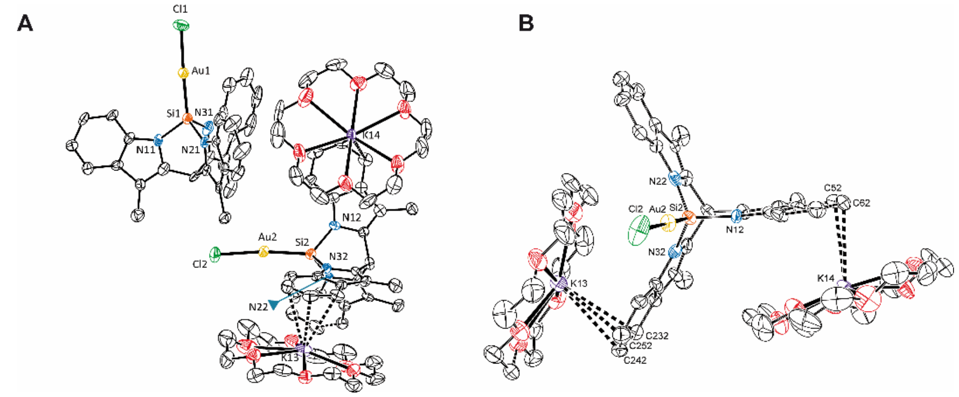

Figure S2. A) Molecular structure of **2** in the crystal (displacement ellipsoids are drawn at the 50% probability level.). C-H hydrogen atoms and non-coordinated solvent are omitted for clarity. Only the major component of the disordered crown ether is shown. B)Interaction between the K cations and the indole groups of the Au complex in **2** (Ellipsoids are drawn at the 50% probability level). Depicted is only the Au complexes which has these interactions. The other independent Au complex does not have these interactions and is omitted in the drawing. C-H hydrogen atoms and disordered solvent molecules are omitted for clarity.

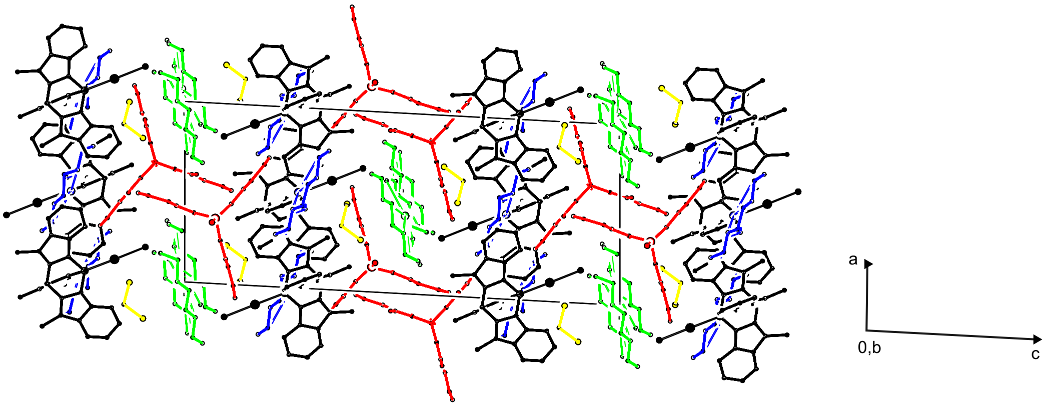

Figure S3. Packing of **2** in the crystal. The two independent Au complexes are drawn in black and red, the two K[18-crown-6] complexes in green and blue. Severely disordered solvent molecules are symbolized in yellow.

$C_{40}H_{46}AuClKN_3O_6Si$ + disordered solvent, Fw = 964.40[*], colourless needle, 0.30 × 0.05 × 0.03 mm^3, monoclinic, $P2_1/n$ (no. 14), a = 12.4909(9), b = 23.7925(11), c = 30.1402(16) Å, β = 93.071(2)°, V = 8944.5(9) Å^3, Z = 8, D_x = 1.432 g/cm^3[*], μ = 3.51 mm^{-1}[*]. The diffraction experiment was performed on a Bruker Kappa ApexII diffractometer with sealed tube and Triumph monochromator (λ = 0.71073 Å) at a temperature of 150(2) K up to a resolution of $(\sin\theta/\lambda)_{max}$ = 0.61 Å^{-1}. The Eval15 software[6] was used for the intensity integration. A numerical absorption correction and scaling was performed with SADABS[7] (correction range 0.26-0.83). A total of 82292 reflections was measured, 16659 reflections were unique (R_{int} = 0.137), 9682 reflections were observed [I>2σ(I)]. The structure was solved with Patterson superposition methods using SHELXT.[8] Structure refinement was performed with SHELXL-2018[9] on F^2 of all reflections. The crystal structure contains solvent accessible voids (1186 Å^3 / unit cell) filled with disordered CH_2Cl_2 solvent molecules. Their contribution to the structure factors was taken into account using the SQUEEZE algorithm[10] resulting in 408 electrons / unit cell. Non-hydrogen atoms were refined freely with anisotropic displacement parameters. Hydrogen atoms were introduced in calculated positions and refined with a riding model. 961 Parameters were refined with no restraints. R1/wR2 [I > 2σ(I)]: 0.0632 / 0.1403. R1/wR2 [all refl.]: 0.1277 / 0.1678. S = 0.970. Residual electron density between -2.32 and 2.53 e/Å^3. Geometry calculations and checking for higher symmetry was performed with the PLATON program.[11]

[*] Derived values do not contain the contribution of the disordered solvent molecules.

4.2. X-ray crystal structure determination of [(tmim)Si]Au]$^-$[(18-crown-6)K]$^+$ (3)

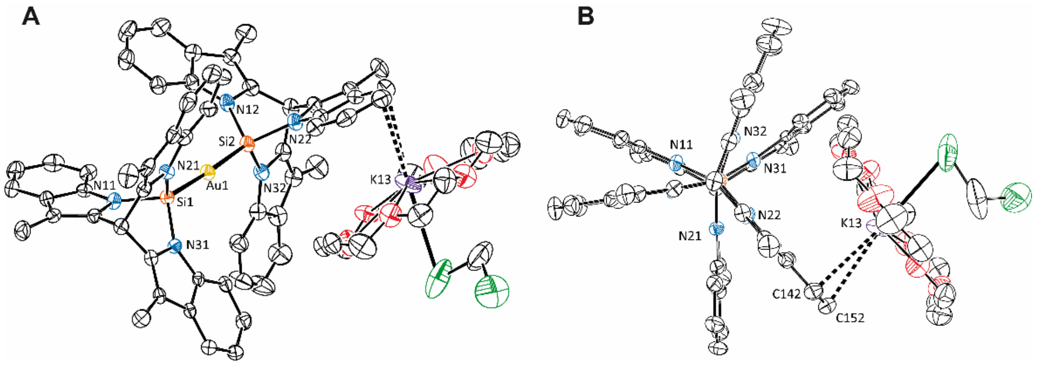

Figure S4. Molecular structure of **3** in the crystal (displacement ellipsoids are drawn at the 50% probability level.). C-H hydrogen atoms and non-coordinated solvent are omitted for clarity. Only the major component of the disordered crown ether is shown.

$[C_{13}H_{26}Cl_2KO_6][C_{56}H_{44}AuN_6Si_2] \cdot 2CH_2Cl_2$, Fw = 1612.30, colourless needle, 0.27 × 0.06 × 0.04 mm^3, monoclinic, $P2_1/c$ (no. 14), a = 12.45218(19), b = 26.6760(6), c = 21.7347(4) Å, β = 90.262(1)°, V = 7219.6(2) Å^3, Z = 4, D_x = 1.483 g/cm^3, μ = 2.41 mm^{-1}. The diffraction experiment was performed on a Bruker Kappa ApexII diffractometer with sealed tube and Triumph monochromator (λ = 0.71073 Å) at a temperature of 150(2) K up to a resolution of $(\sin\theta/\lambda)_{max}$ = 0.65 Å^{-1}. The Eval15 software[6] was used for the intensity integration. A numerical absorption correction and scaling was performed with SADABS[7] (correction range 0.68-0.98). A total of 70305 reflections was measured, 16586 reflections were unique (R_{int} = 0.056), 12085 reflections were observed [I>2σ(I)]. The structure was solved with Patterson superposition methods using SHELXT.[8] Structure refinement was performed with SHELXL-2018 on F^2 of all reflections. Non-hydrogen atoms were refined freely with anisotropic displacement parameters. The crown ether

ligand and one of the CH_2Cl_2 solvent molecules were refined with disorder models. The disorder of the solvent was not fully resolved. Hydrogen atoms were introduced in calculated positions and refined with a riding model. 1019 Parameters were refined with 1109 restraints (distances, angles and displacement parameters of the disordered groups). R1/wR2 [$I > 2\sigma(I)$]: 0.0455 / 0.1108. R1/wR2 [all refl.]: 0.0724 / 0.1225. S = 1.040. Residual electron density between -1.43 and 2.18 e/Å^3. Geometry calculations and checking for higher symmetry was performed with the PLATON program.[11]

4.3. X-ray crystal structure determination of [(tmim)SiAu)$_2$Cl]$^-$[K]$^+$ (4)

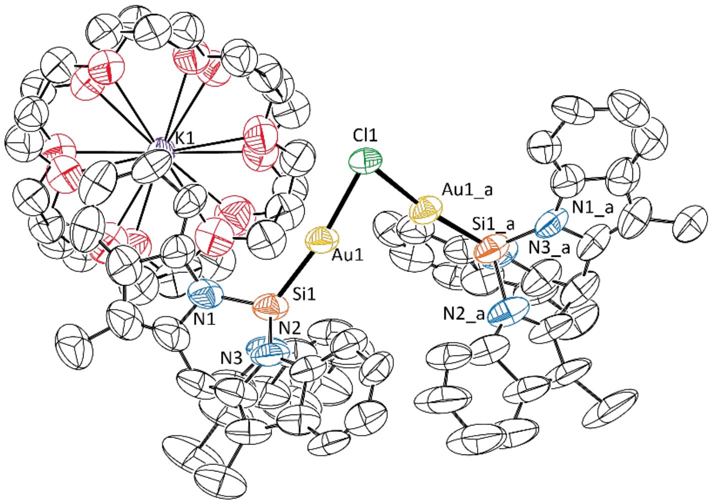

Figure S5. Molecular structure of **4** in the crystal (displacement ellipsoids are drawn at the 50% probability level.). C-H hydrogen atoms and non-coordinated solvent are omitted for clarity.

[$C_{12}H_{24}KO_6$][$C_{56}H_{44}Au_2ClN_6Si_2$] · 1.26($CH_2Cl_2$), Fw = 1697.80, red plate, 0.28 × 0.26 × 0.03 mm^3, orthorhombic, $P2_12_12$ (no. 18), a = 12.4438(5), b = 16.1204(9), c = 17.9652(7) Å, V = 3603.8(3) Å^3, Z = 2, D_x = 1.565 g/cm^3, μ = 10.01 mm^{-1}. The diffraction experiment was performed on a Bruker Proteum diffractometer with rotating anode and Helios optics (λ = 1.54184 Å) at a temperature of 150(2) K up to a resolution of $(\sin\theta/\lambda)_{max}$ = 0.57 Å^{-1}. The diffraction pattern is characterized by strong diffuse streaks in the direction of *hkl*=(0,0,1). The Eval15 software[6] was used for the intensity integration. The prediction of reflection profiles involved an isotropic mosaicity of 1.1° and a mica contour expansion of 0.3 in the direction of *hkl*=(0,0,1). A multi-scan absorption correction and scaling was performed with SADABS[7] (correction range 0.17-0.46). A total of 20081 reflections was measured, 5681 reflections were unique (R_{int} = 0.063), 4926 reflections were observed [$I>2\sigma(I)$]. The structure was solved with Patterson superposition methods using SHELXT.[8] Structure refinement was performed with SHELXL-2018[9] on F^2 of all reflections. Non-hydrogen atoms were refined freely with anisotropic displacement parameters. Hydrogen atoms were introduced in calculated positions and refined with a riding model. 499 Parameters were refined with 537 restraints (distances and angles in the crown ether and the partially occupied CH_2Cl_2, displacement parameters of all organic residues). R1/wR2 [$I > 2\sigma(I)$]: 0.0924 / 0.2310. R1/wR2 [all refl.]: 0.0999 / 0.2382. S = 1.136. Flack parameter[12] x=0.13(4) from a refinement as an inversion twin. Residual electron density between -0.96 and 4.24 e/Å^3 (in proximity of the heavy atom Au). Geometry calculations and checking for higher symmetry was performed with the PLATON program.[11]

4.4. X-ray crystal structure determination of [(tmim)SiGeCl]$^-$[K]$^+$ (5)

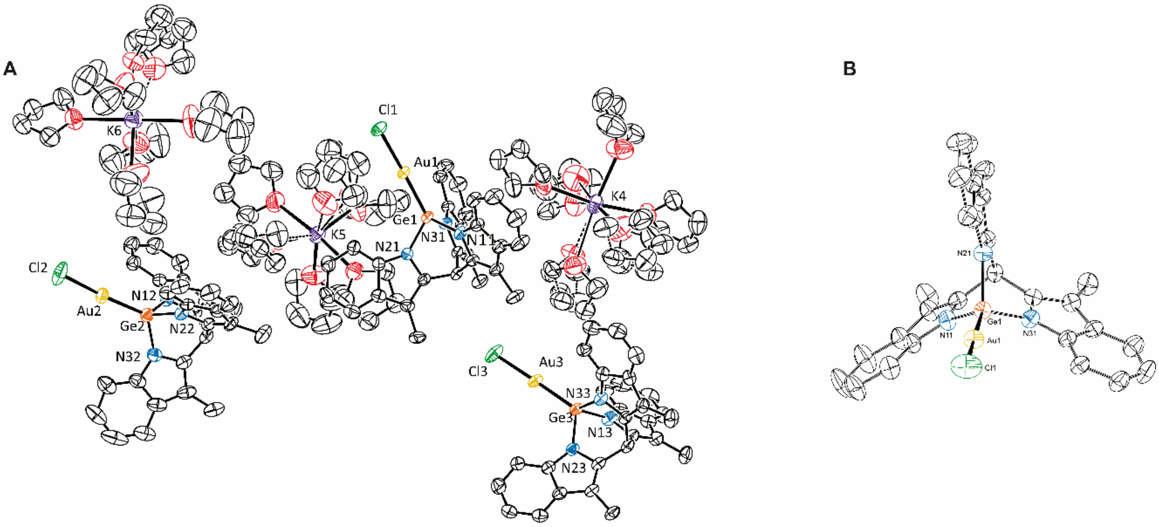

Figure S6. Molecular structure of **5** in the crystal (displacement ellipsoids are drawn at the 50% probability level.). C-H hydrogen atoms and non-coordinated solvent are omitted for clarity.

$[C_{24}H_{48}KO_6][C_{28}H_{22}AuClGeN_3] \cdot 1/3(C_4H_8O)$, Fw = 1201.25, colourless block, 0.36 × 0.29 × 0.19 mm^3, triclinic, P1 (no. 1), a = 12.15990(19), b = 12.6864(3), c = 29.8405(4) Å, α = 89.446(1), β = 87.008(1), γ = 62.434(1)°, V = 4074.68(12) Å^3, Z = 3, D_x = 1.469 g/cm^3, μ = 3.43 mm^{-1}. The diffraction experiment was performed on a Bruker Kappa ApexII diffractometer with sealed tube and Triumph monochromator (λ = 0.71073 Å) at a temperature of 150(2) K up to a resolution of $(\sin \theta/\lambda)_{max}$ = 0.65 Å^{-1}. The Eval15 software[6] was used for the intensity integration. A numerical absorption correction and scaling was performed with SADABS[7] (correction range 0.40-0.66). A total of 122438 reflections was measured, 37468 reflections were unique (R_{int} = 0.035), 34421 reflections were observed [$I>2\sigma(I)$]. The structure was solved with Patterson superposition methods using SHELXT.[8] Structure refinement was performed with SHELXL-2018[9] on F^2 of all reflections. Non-hydrogen atoms were refined freely with anisotropic displacement parameters. Six of the coordinated THF molecules were refined with disorder models. The disorder could not be fully resolved. Hydrogen atoms were introduced in calculated positions and refined with a riding model. 2042 Parameters were refined with 7498 restraints (distances, angles and displacement parameters of the THF molecules). R1/wR2 [$I > 2\sigma(I)$]: 0.0329 / 0.0851. R1/wR2 [all refl.]: 0.0375 / 0.0875. S = 1.028. Flack parameter[12] x=-0.007(4) from a refinement as an inversion twin. Residual electron density between -0.68 and 1.67 e/Å^3. Geometry calculations and checking for higher symmetry was performed with the PLATON program.[11]

4.5. X-ray crystal structure determination of $[(tmim)Ge]Au]^-[K]^+$ (6)

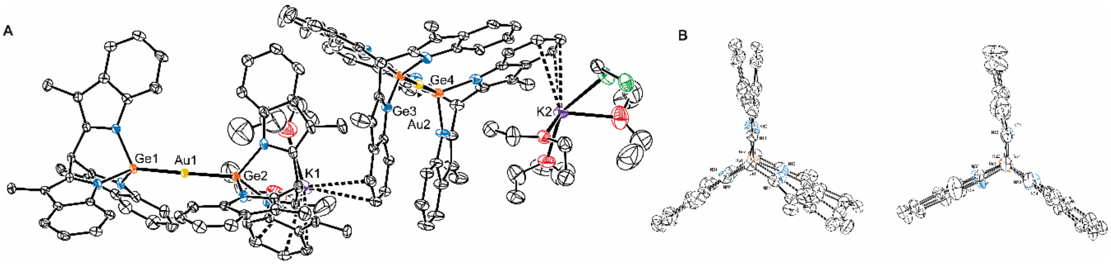

Figure S7. A) Interaction between the K cations and the indole groups of the Au complexes in **6**. Atom K1 is bridging the indole moieties of two Au complexes, K2 is connected to only one Au complex. H atoms and non-coordinated solvent molecules are omitted for clarity. Only the major disorder component is shown. Ellipsoids are at the 30% probability level.B) Displacement ellipsoids are drawn at the 50% probability level.

$C_{56}H_{44}AuGe_2KN_6 \cdot 2.89(C_4H_{10}O) \cdot 1.11(CH_2Cl_2)$, Fw = 1490.72, colourless block, 0.44 × 0.17 × 0.14 mm^3, triclinic, $P\overline{1}$ (no. 2), a = 16.1510(6), b = 17.8634(6), c = 23.3415(10) Å, α = 79.711(1), β = 83.572(1), γ = 81.384(2)°, V = 6526.0(4) $Å^3$, Z = 4, D_x = 1.517 g/cm^3, μ = 3.36 mm^{-1}. The diffraction experiment was performed on a Bruker Kappa ApexII diffractometer with sealed tube and Triumph monochromator (λ = 0.71073 Å) at a temperature of 150(2) K up to a resolution of $(\sin \theta/\lambda)_{max}$ = 0.65 $Å^{-1}$. The crystal appeared to be cracked into two fragments with an angle of 6.9° between them. Consequently, two orientation matrices were used for the integration with the Eval15 software[6] resulting in a HKLF-5 file. A multi-scan absorption correction and scaling was performed with TWINABS[13] (correction range 0.51-0.75). A total of 113089 reflections was measured, 29991 reflections were unique (Rint = 0.047), 25386 reflections were observed [I>2σ(I)]. The structure was solved with Patterson superposition methods using SHELXT.[8] Structure refinement was performed with SHELXL-2018[9] on F2 of all reflections. Non-hydrogen atoms were refined freely with anisotropic displacement parameters. Partially and fully occupied solvent molecules were refined with disorder models. The disorder could not be fully resolved. Hydrogen atoms were introduced in calculated positions and refined with a riding model. 1619 Parameters were refined with 863 restraints (distances, angles and displacement parameters of the disordered molecules). R1/wR2 [I > 2σ(I)]: 0.0410 / 0.1105. R1/wR2 [all refl.]: 0.0511 / 0.1172. S = 1.032. Batch scale factor BASF=0.1006(6). Residual electron density between -1.73 and 2.66 e/Å3. Geometry calculations and checking for higher symmetry was performed with the PLATON program.[12]

5. DFT studies

5.1. Computational methods

Calculations were performed using Gaussian16, Revision C.01. [14] Geometry optimizations were carried out in vacuum at the B3LYP-GD3 level of theory, the 6-31g(d,p) basis set was used for all the small atoms and LANL2DZ for Au. Frequency analyses on all stationary points were used to ensure that they are minima (no imaginary frequency). Moreover, the optimized structures were compared to the corresponding crystal structure to assess the validity of the method used. NBO analysis[15] was performed with the NBO6 program and was carried out in vacuum at the B3LYP-GD3 level of theory, the 6-311+G(d,p) basis set was used for all the small atoms and LANL2TZ(f)[16]or Au.

5.2. NBO Calculations

NBO analyses of the Si and Ge monoligated and bisligated anionic complexes **2, 3, 5**, and **6** showed that the linear coordination at Au is best described as a 3c/4e- bonding situation (ω type bond) consisting of the resonances structures: $\boldsymbol{L_1} - \boldsymbol{Au} : \boldsymbol{L_2} \leftrightarrow \boldsymbol{L_1}\!: \boldsymbol{Au} - \boldsymbol{L_2}$. It is characterized by a high occupancy of the $\sigma_{\boldsymbol{Au-L1}}$ bonding orbital, a relatively low occupancy of the $\boldsymbol{LP_{L_2}}$ lone

pair, and high $LP_{L_2} \rightarrow \sigma^*_{Au-L_1}$ second order perturbation energy (See table). For all complexes, the σ_{Au-L1} orbital is strongly polarized toward **E** (Si or Ge), even though Au is more electronegative than Si and Ge, illustrating the stabilizing effect of the tmim cage on the tetrel lone pair. Moreover, the $LP_{L_2} \rightarrow \sigma^*_{Au-L_1}$ second order perturbation energy is much higher for the bisligated species **3** and **6** compared to the monoligated ones **2** and **5**. This suggests that the elongation of the Au-L1 bond observed in the crystal structures of **3** and **6** compared to **2** and **5** originates from the competition of both tetrel lone-pairs for the accepting Au-centered orbital.

	**2**	**3**	**5**	**6**
**WBI(Au-Cl)**	0.32	-	0.37	-
**WBI(Au-E1)**	0.82	0.56	0.71	0.54
**WBI(Au-E2)**	-	0.56	-	0.54
**q(Au)**	0.24	0.06	0.28	0.21
**q(Cl)**	0.72	-	-0.70	-
**q(E1)**	1.38	1.33	1.24	1.23
**q(E2)**	-	1.34	-	1.23
**q(Au + Cl)**	-0.48	-	-0.42	-
**q(E1 + Au)**	1.62	1.39	1.52	1.44
**q(E2 + Au)**	-	1.40	-	1.44
**E ($LP_{L_2} \rightarrow \sigma^*_{Au-L_1}$) kcal/mol**	74.9	364.6	81.8	0.54

5.3. LUMO's analysis

The phenylacetylene complexes with the neutral (tmim)SiAu and (tmim)GeAu were investigated computationally. The LUMO in both cases is mostly an alkyne π* orbital with a bonding component to Au, and is most likely the orbital involved in nucleophilic attack. The HOMO-LUMO gap for Ge is significantly smaller (45.4 kcal/mol) compared to Si (55.3 kcal/mol), which is consistent with a higher activity of the Ge compound.

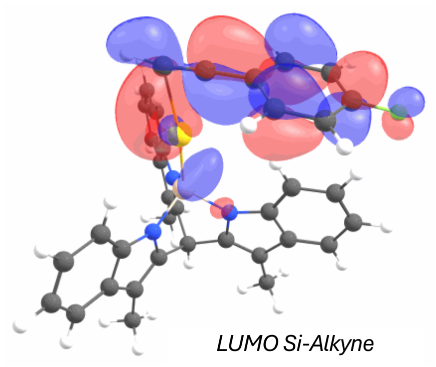

LUMO Si-Alkyne

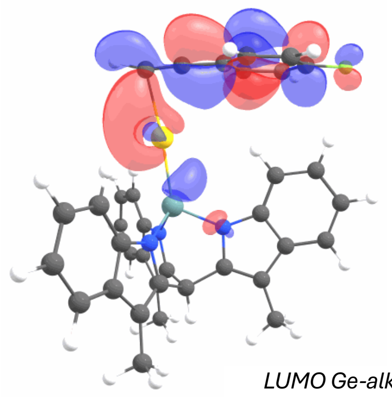

LUMO Ge-alkyne

6. Spectra of isolated compounds

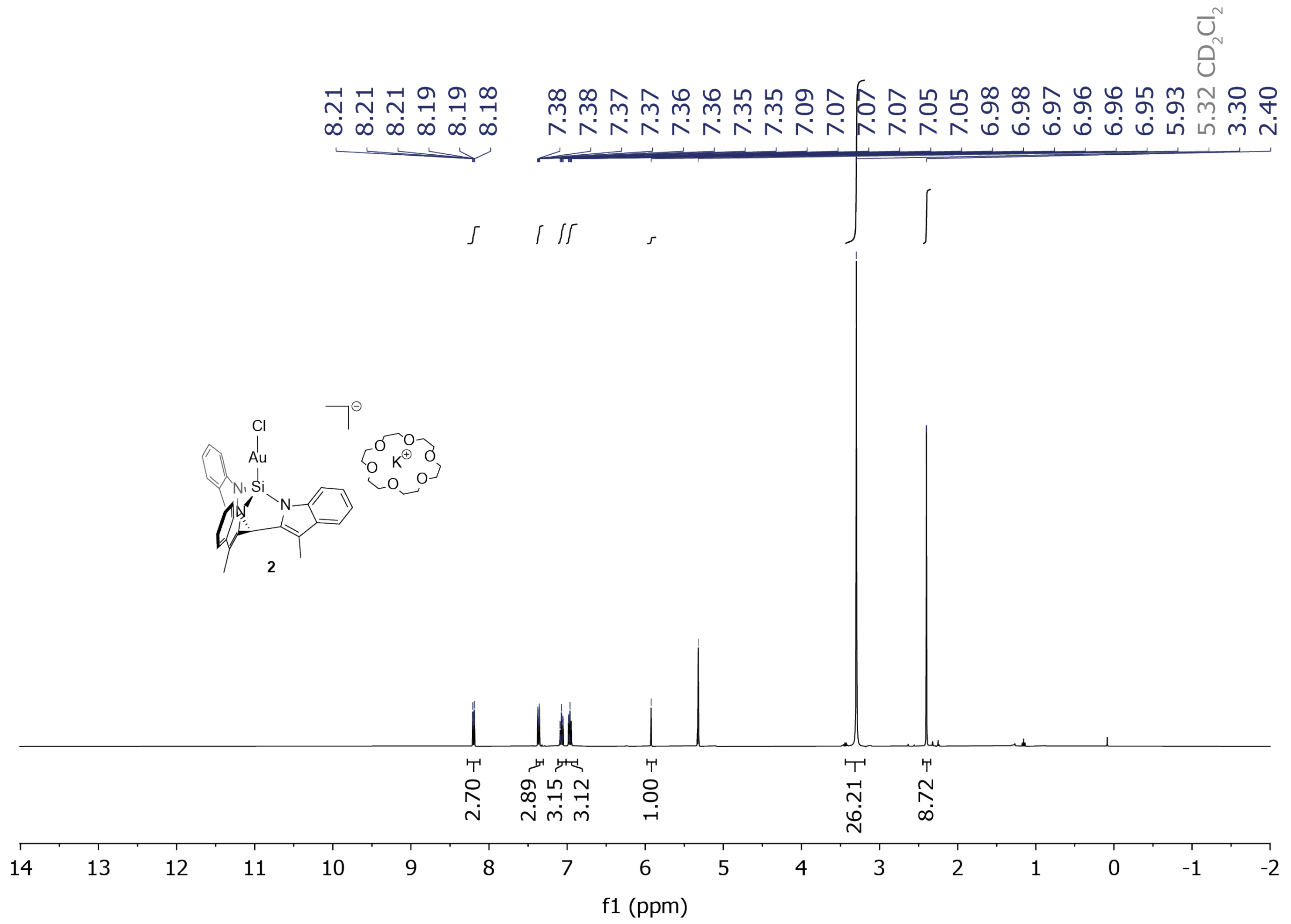

Figure S8. ^{1}H-NMR(400 MHz) spectrum of compound **2** in CD_2Cl_2 at 25°C.

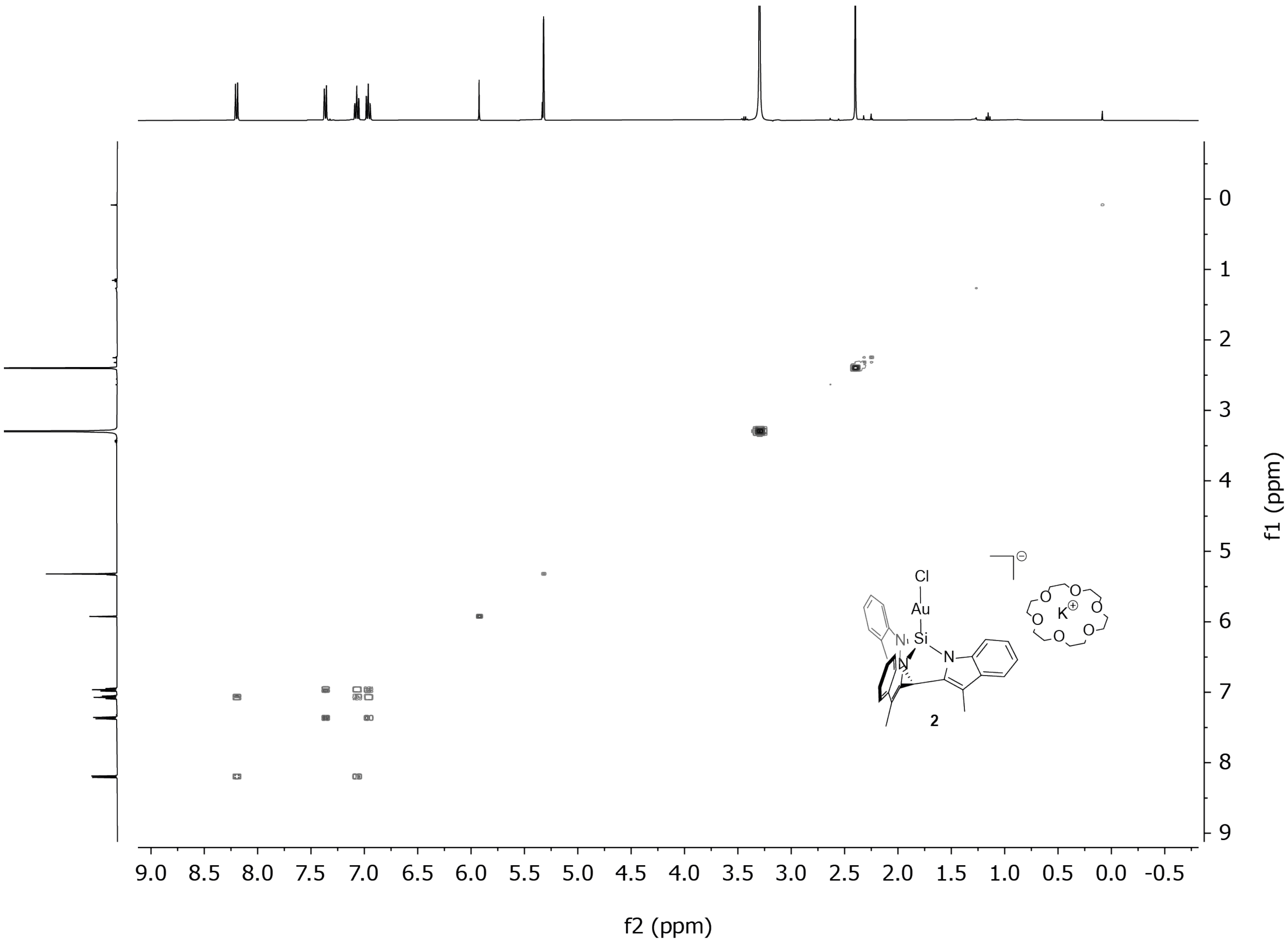

Figure S9. ^{1}H-COSY (400 MHz) spectrum of compound **2** in CD_2Cl_2 at 25°C.

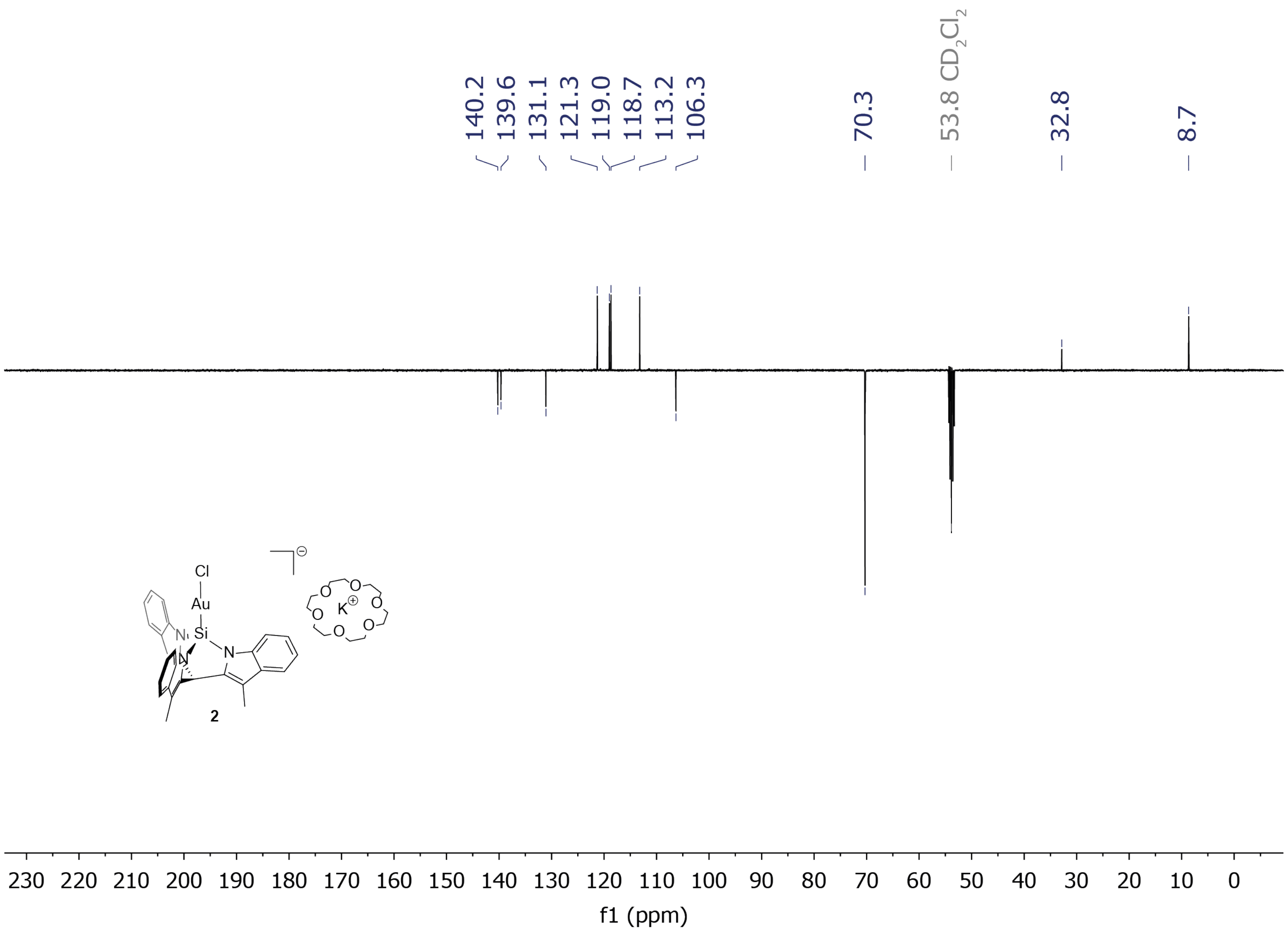

Figure S10. ^{13}C-NMR(101 MHz) spectrum of compound **2** in CD_2Cl_2 at 25°C.

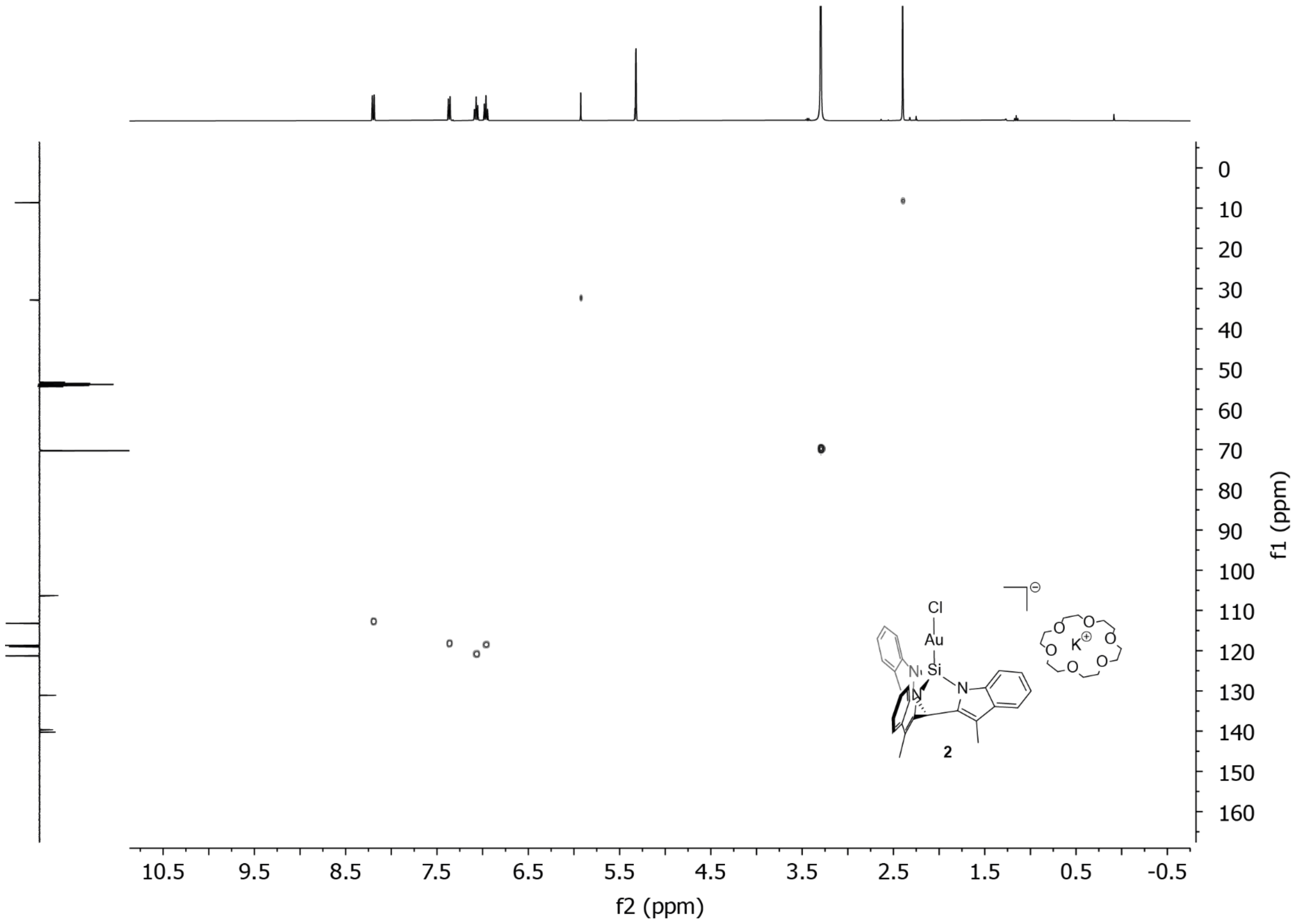

Figure S11. ^{1}H-^{13}C HSQC spectrum of compound **2** in CD_2Cl_2 at 25°C.

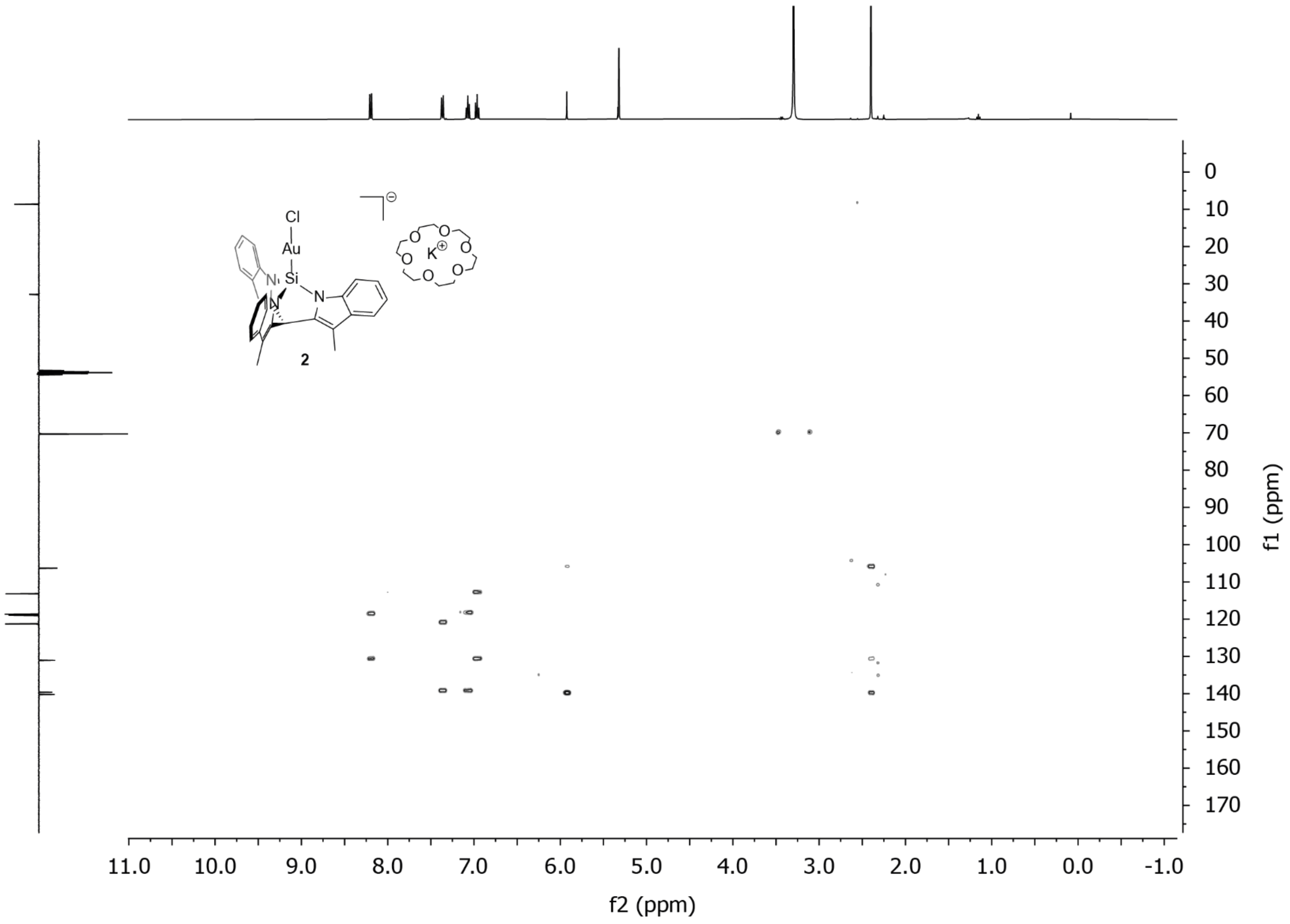

Figure S12. ^{1}H-^{13}C HMBC spectrum of compound **2** in CD_2Cl_2 at 25°C.

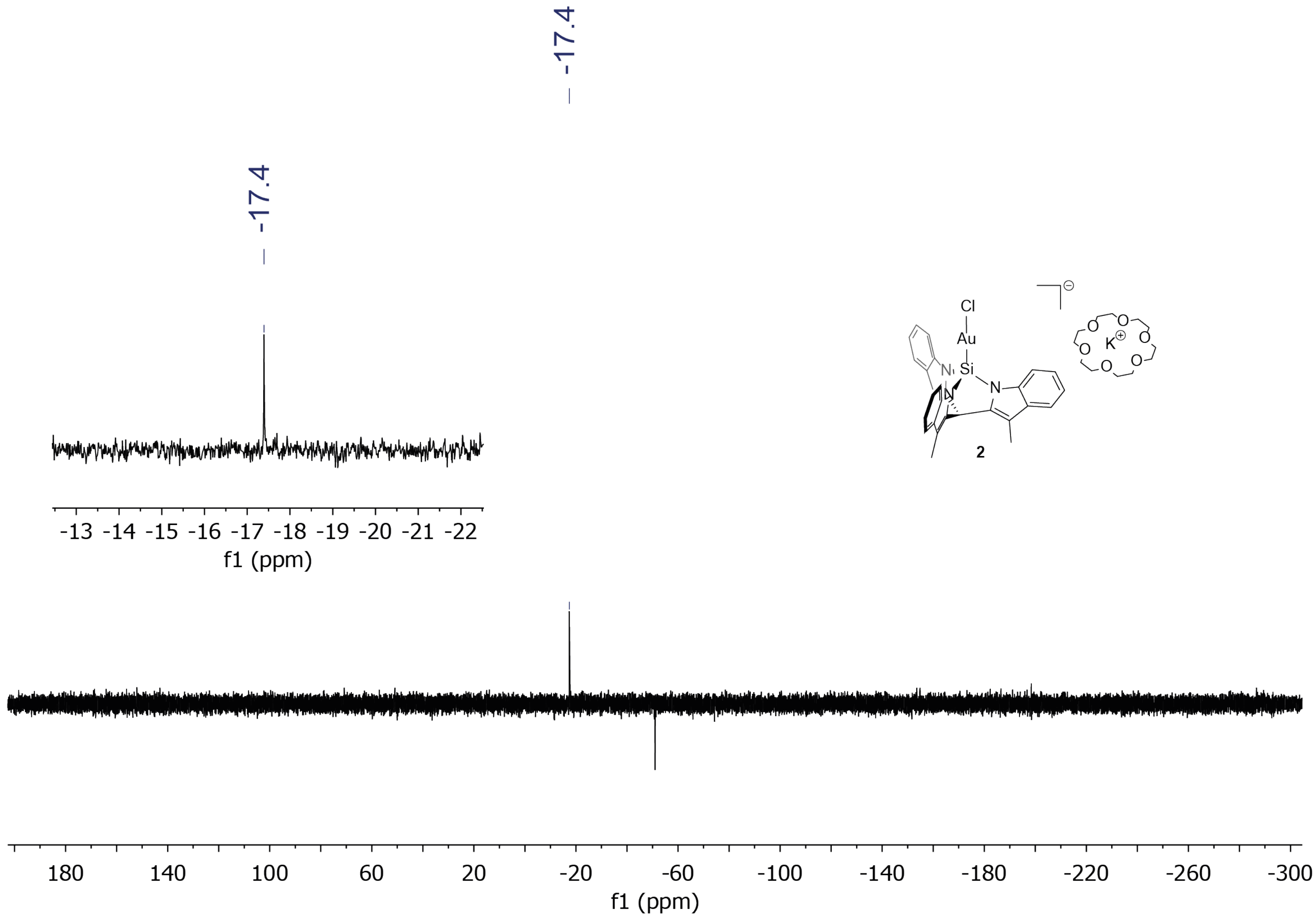

Figure S13. ^{29}Si INEPT Coupled (79 MHz) spectrum of compound **2** in CD_2Cl_2 at 25°C. Peak around -50 ppm: Center glitch artifact at the center of the measurement window.

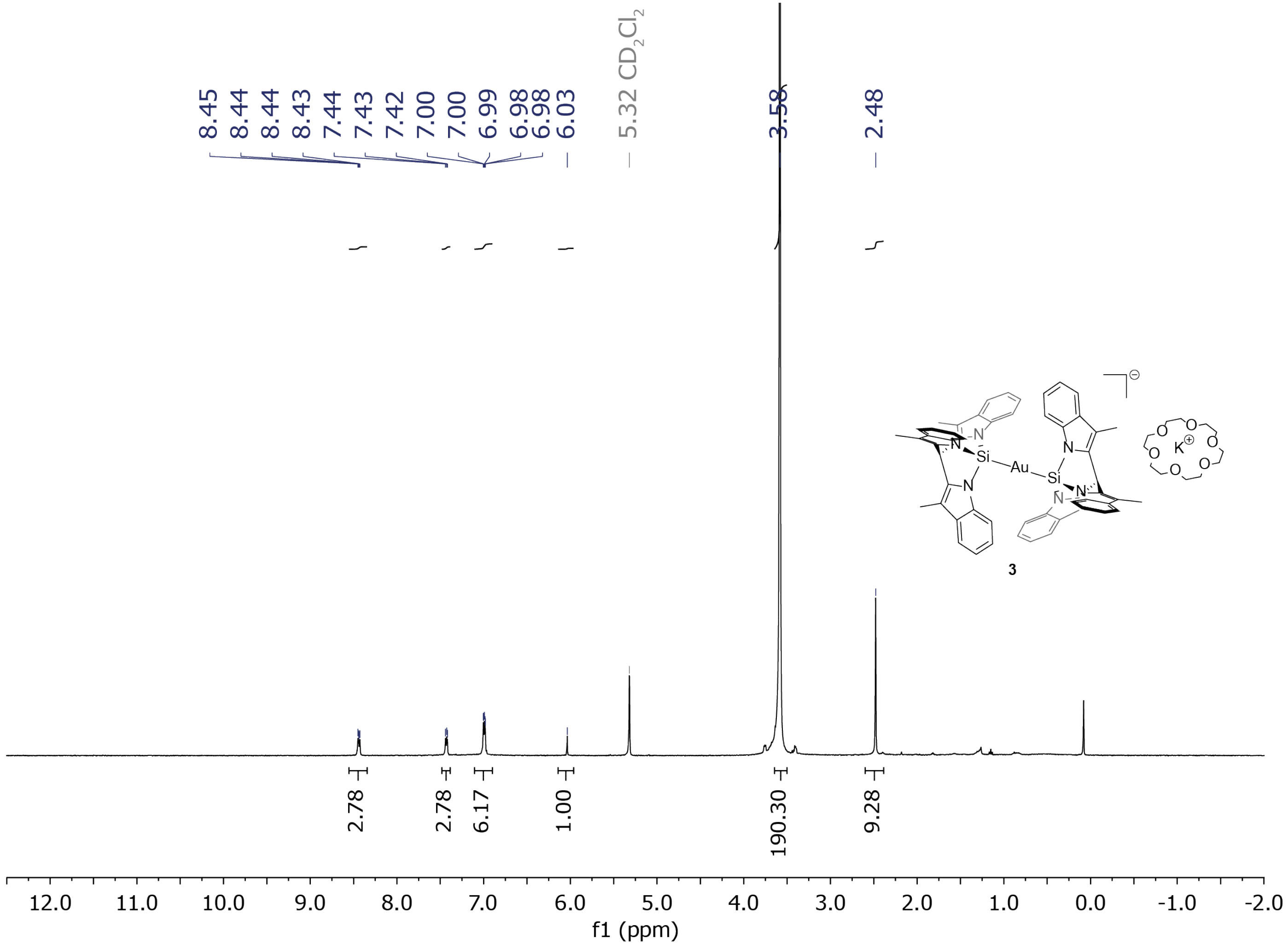

Figure S14. ^{1}H-NMR (400 MHz) spectrum of compound **3** in CD_2Cl_2 at 25°C.

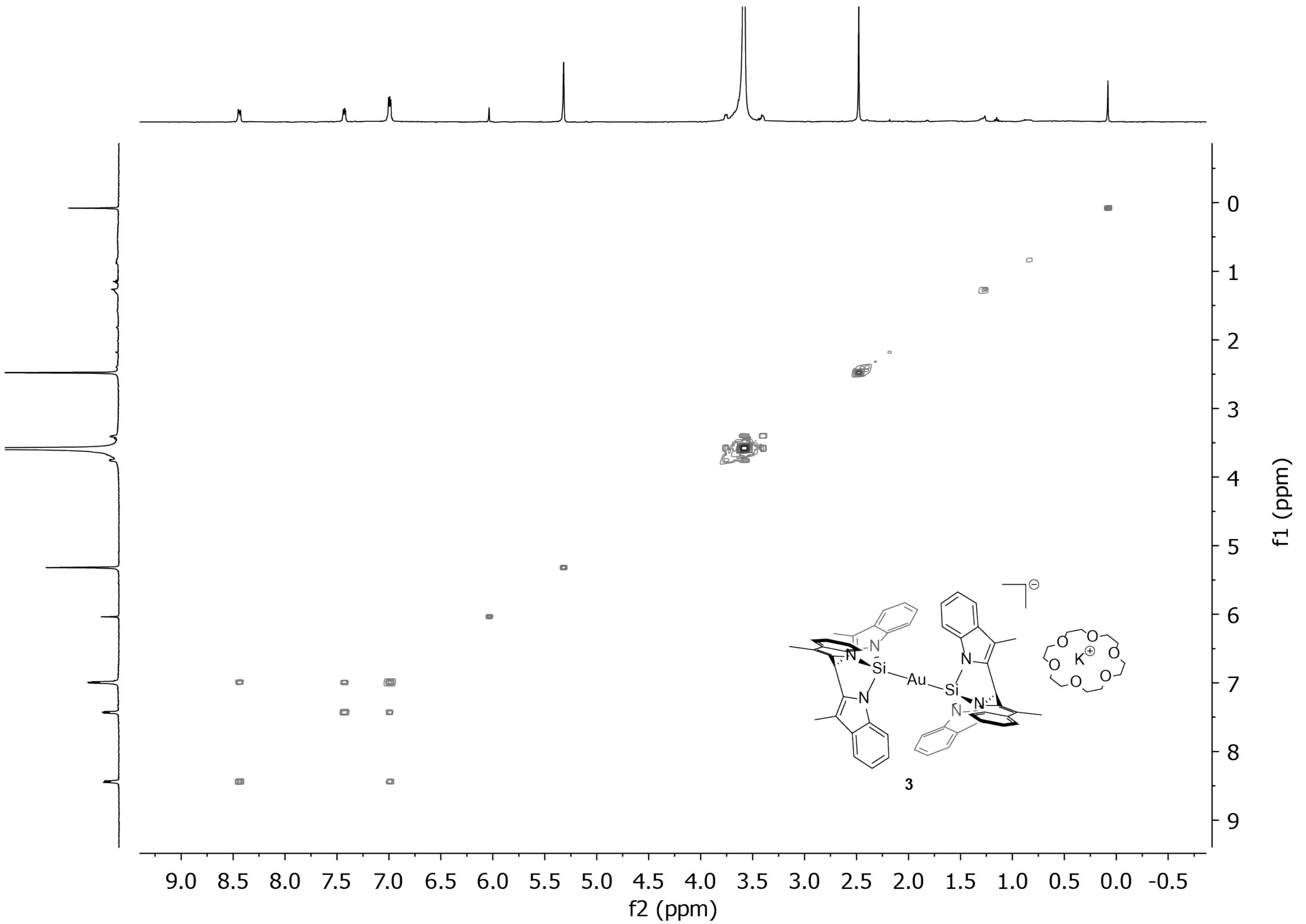

Figure S15. ^{1}H-COSY (400 MHz) spectrum of compound **3** in CD_2Cl_2 at 25°C.

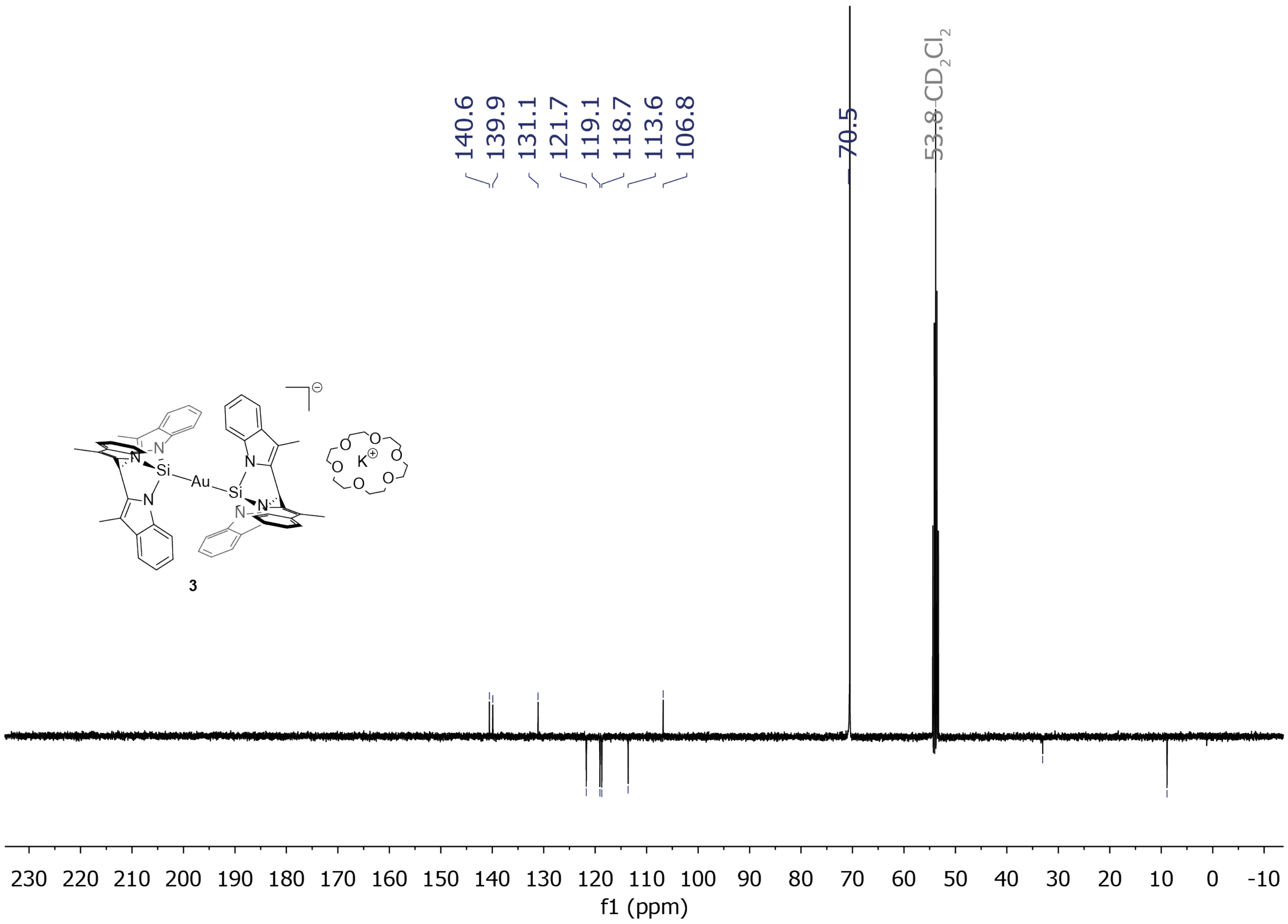

Figure S16. ^{13}C-NMR(101 MHz) spectrum of compound **3** in CD_2Cl_2 at 25°C.

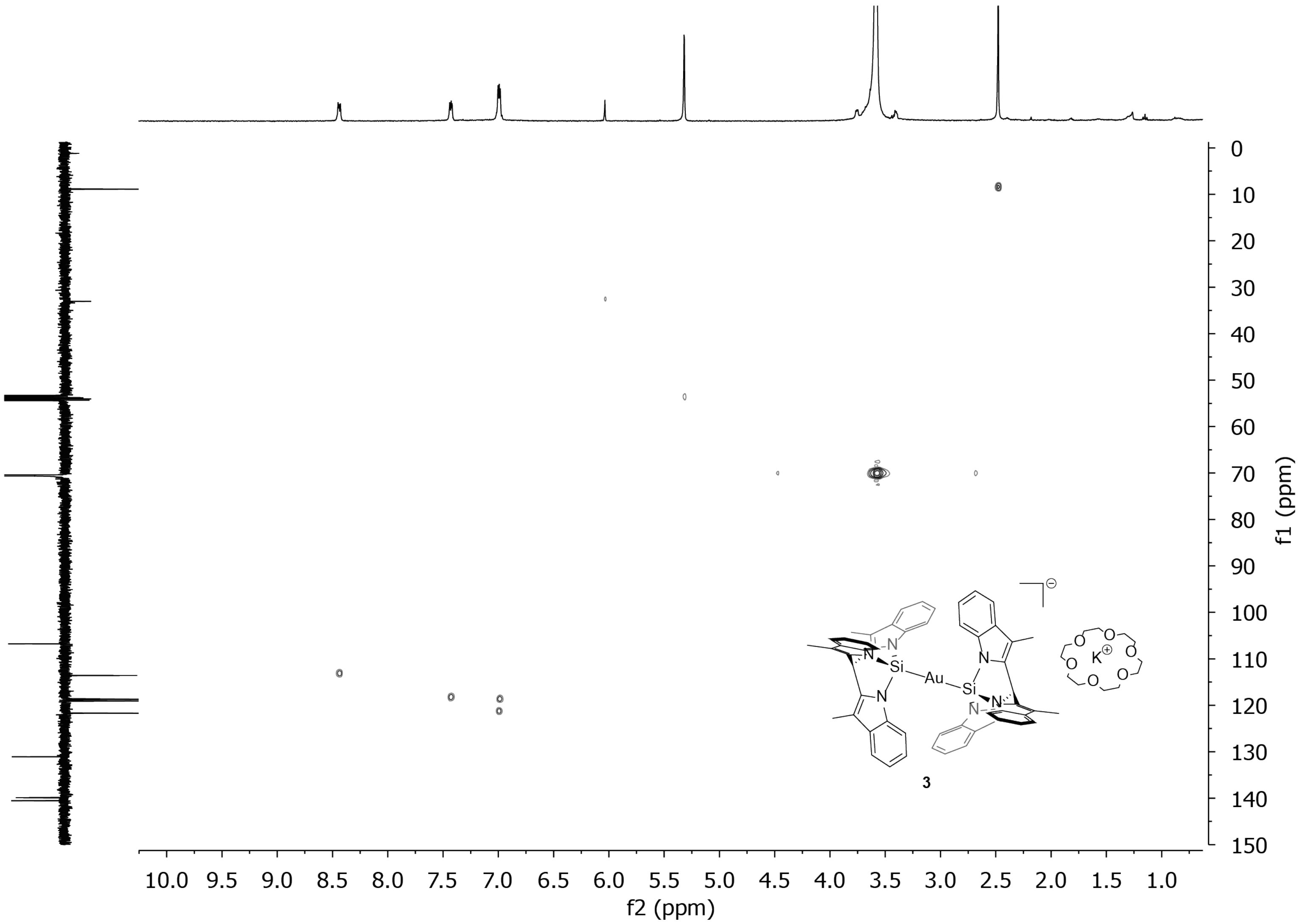

Figure S17. ^{1}H-^{13}C HSQC spectrum of compound **3** in CD_2Cl_2 at 25°C.

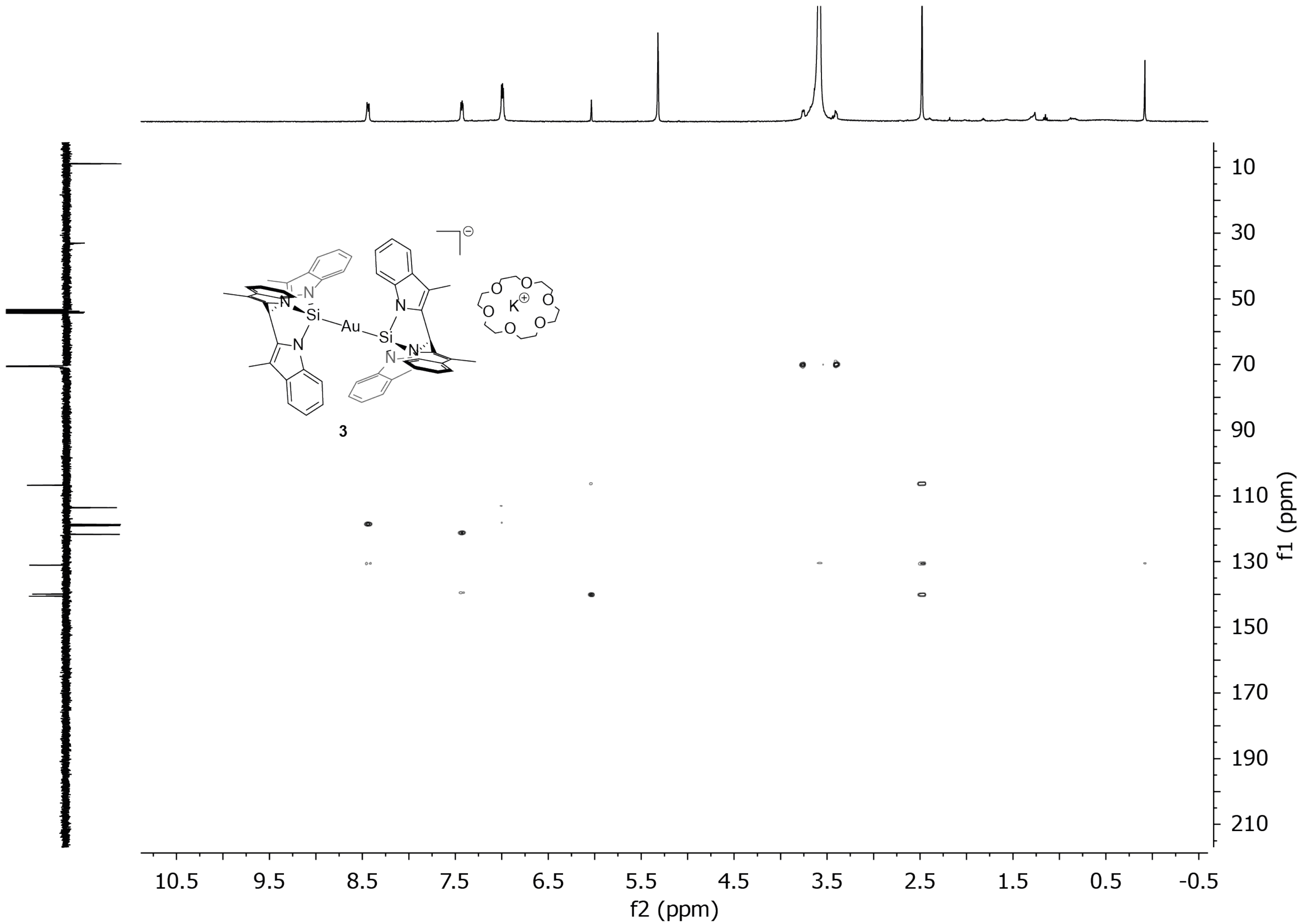

Figure S18. ^{1}H-^{13}C HMBC spectrum of compound **3** in CD_2Cl_2 at 25°C.

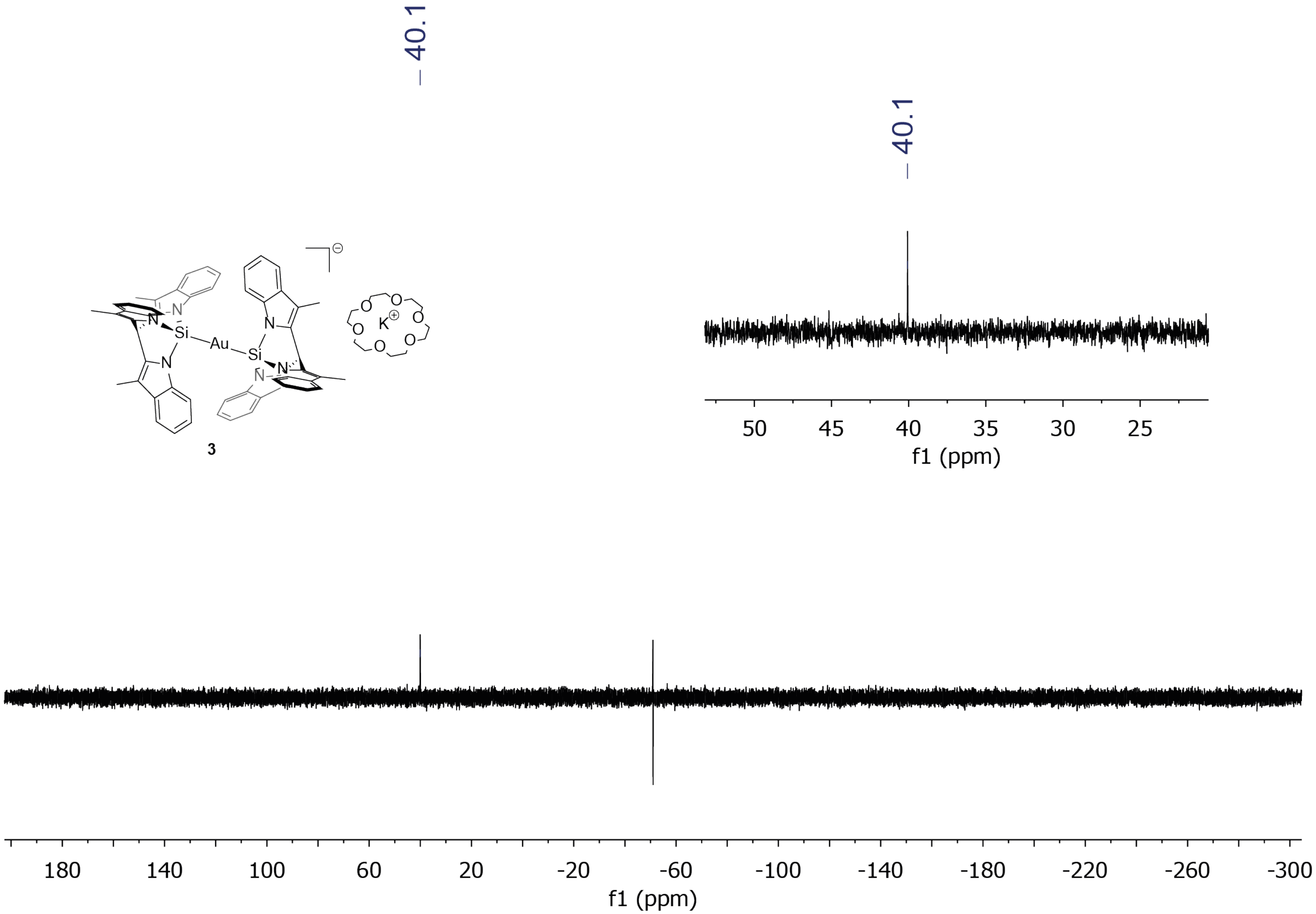

Figure S19. ^{29}Si-NMR (79 MHz) spectrum of compound **3** in CD_2Cl_2 at 25°C. Peak around -50 ppm: Center glitch artifact at the center of the measurement window.

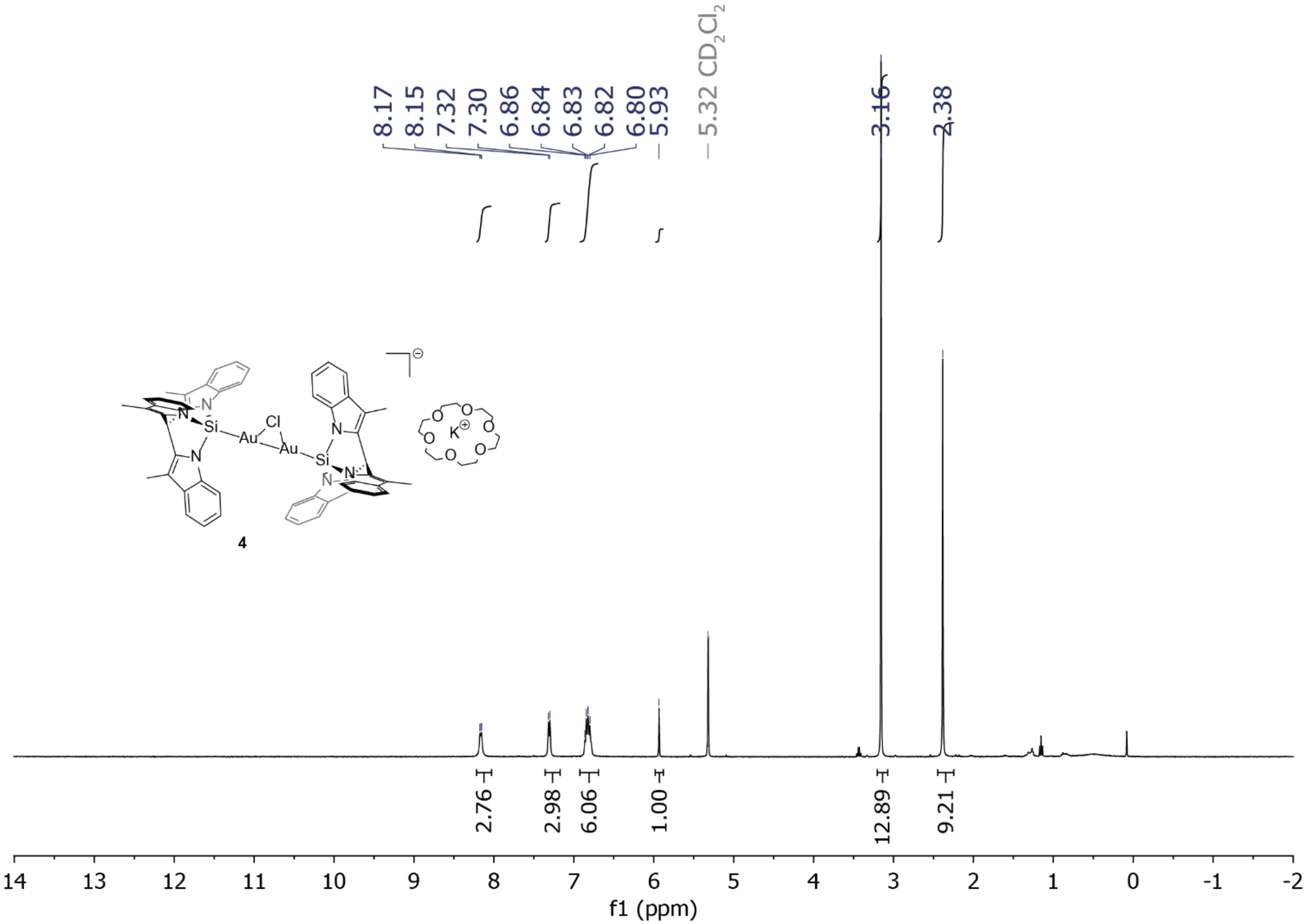

Figure S20. ^{1}H-NMR (400 MHz) spectrum of compound **4** in CD_2Cl_2 at 25°C.

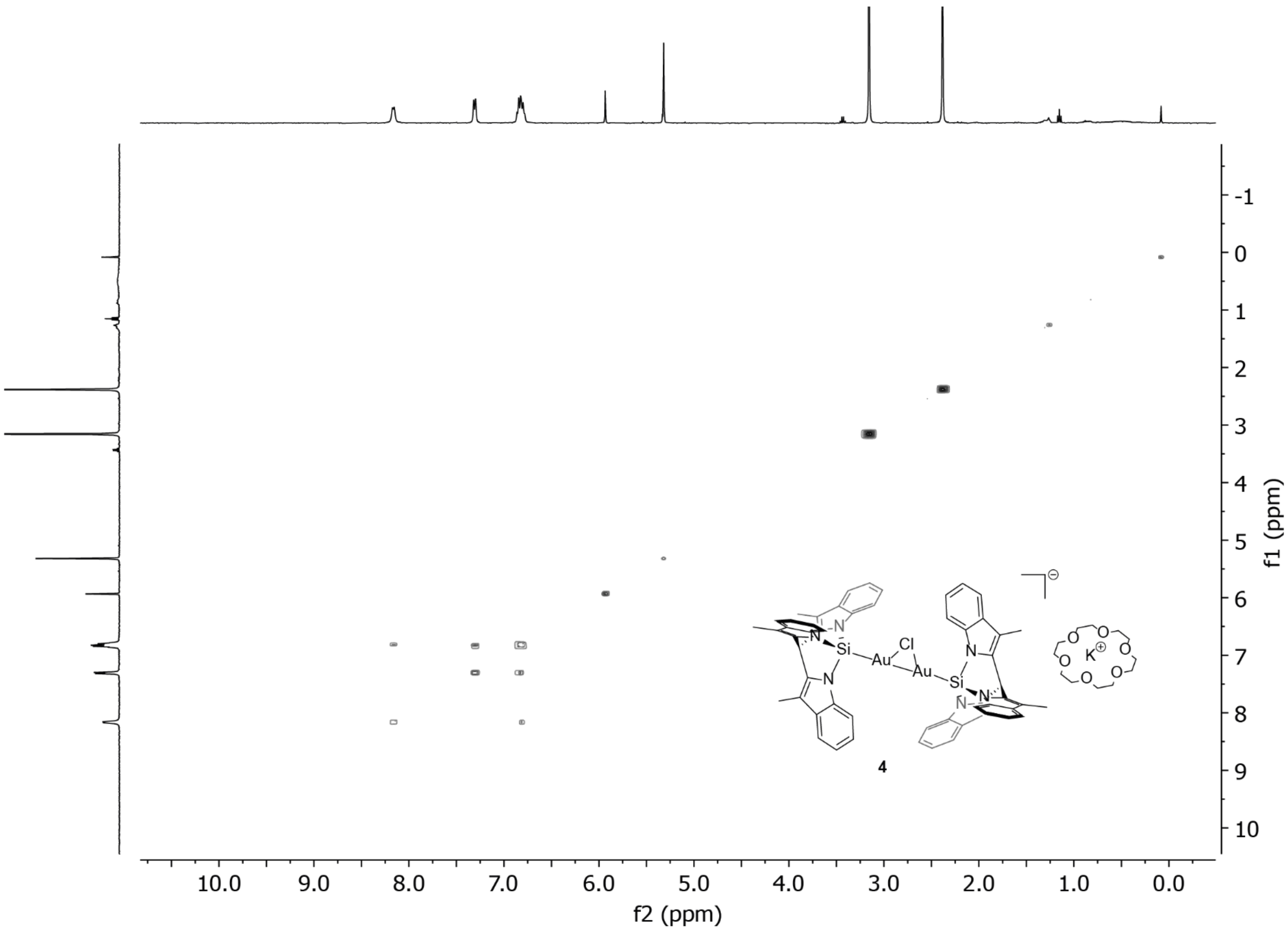

Figure S21. ^{1}H-COSY (400 MHz) spectrum of compound **4** in CD_2Cl_2 at 25°C.

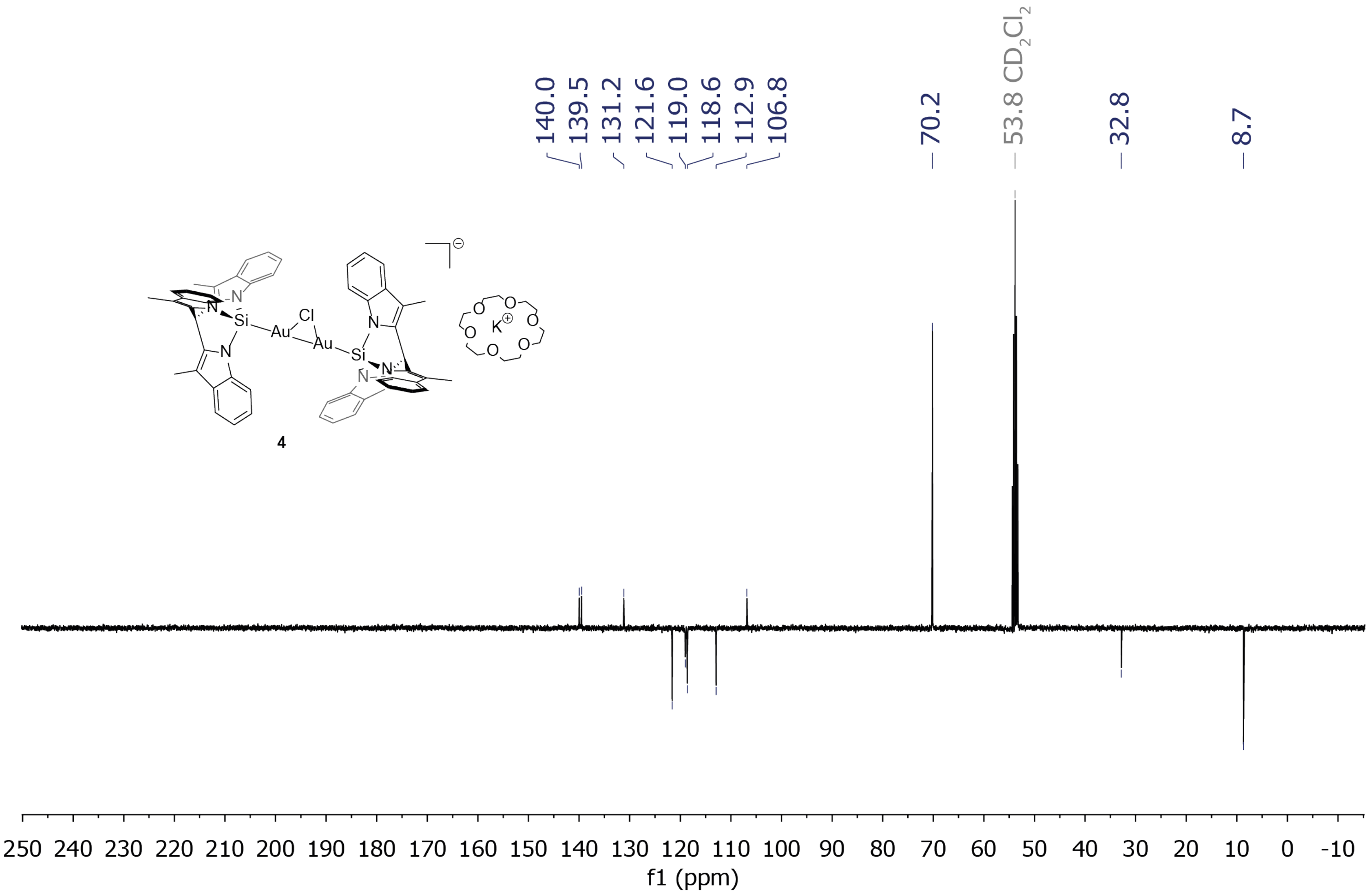

Figure S22. ^{13}C-NMR(101 MHz) spectrum of compound **4** in CD_2Cl_2 at 25°C.

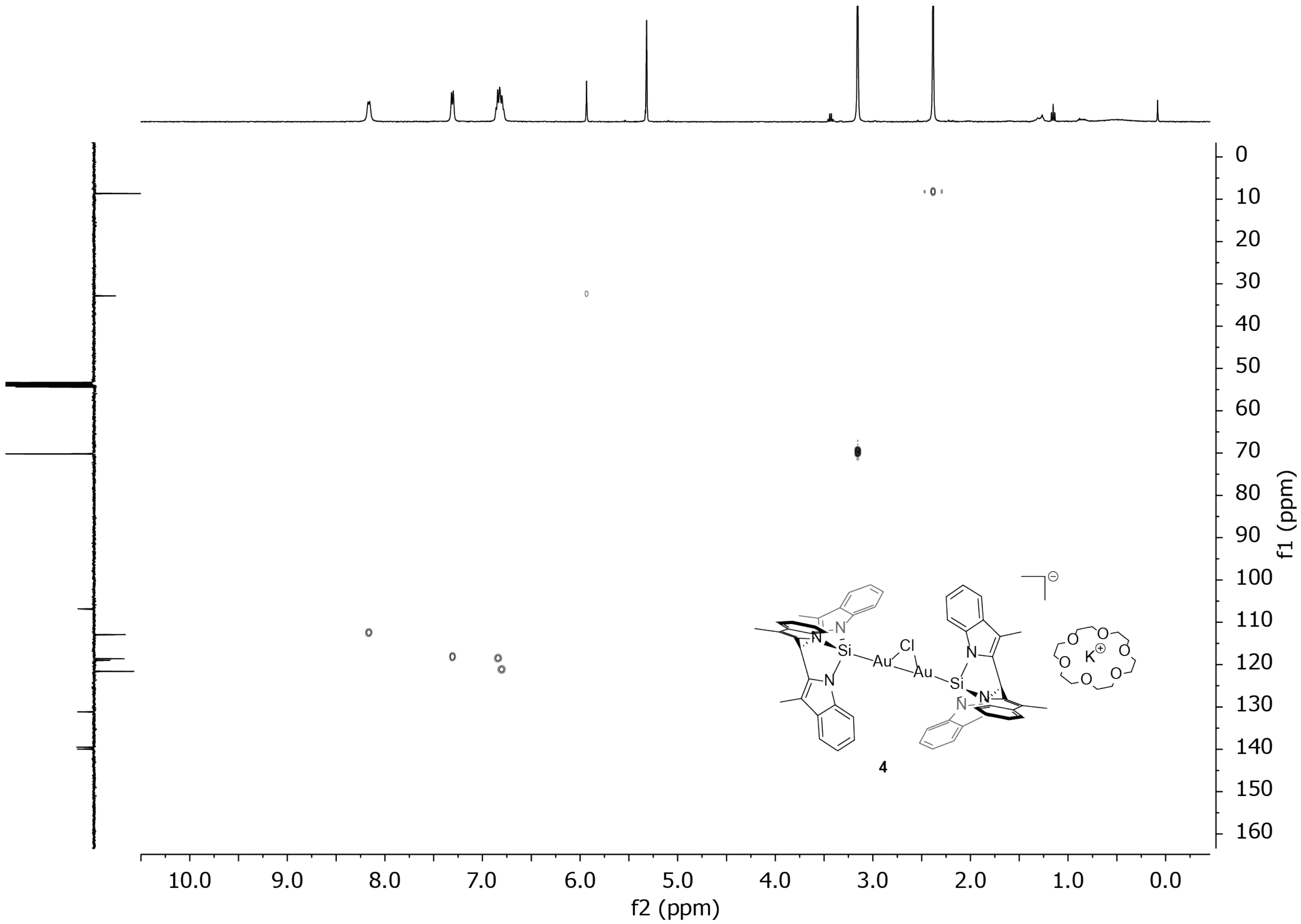

Figure S23. ^{1}H-^{13}C HSQC spectrum of compound **4** in CD_2Cl_2 at 25°C.

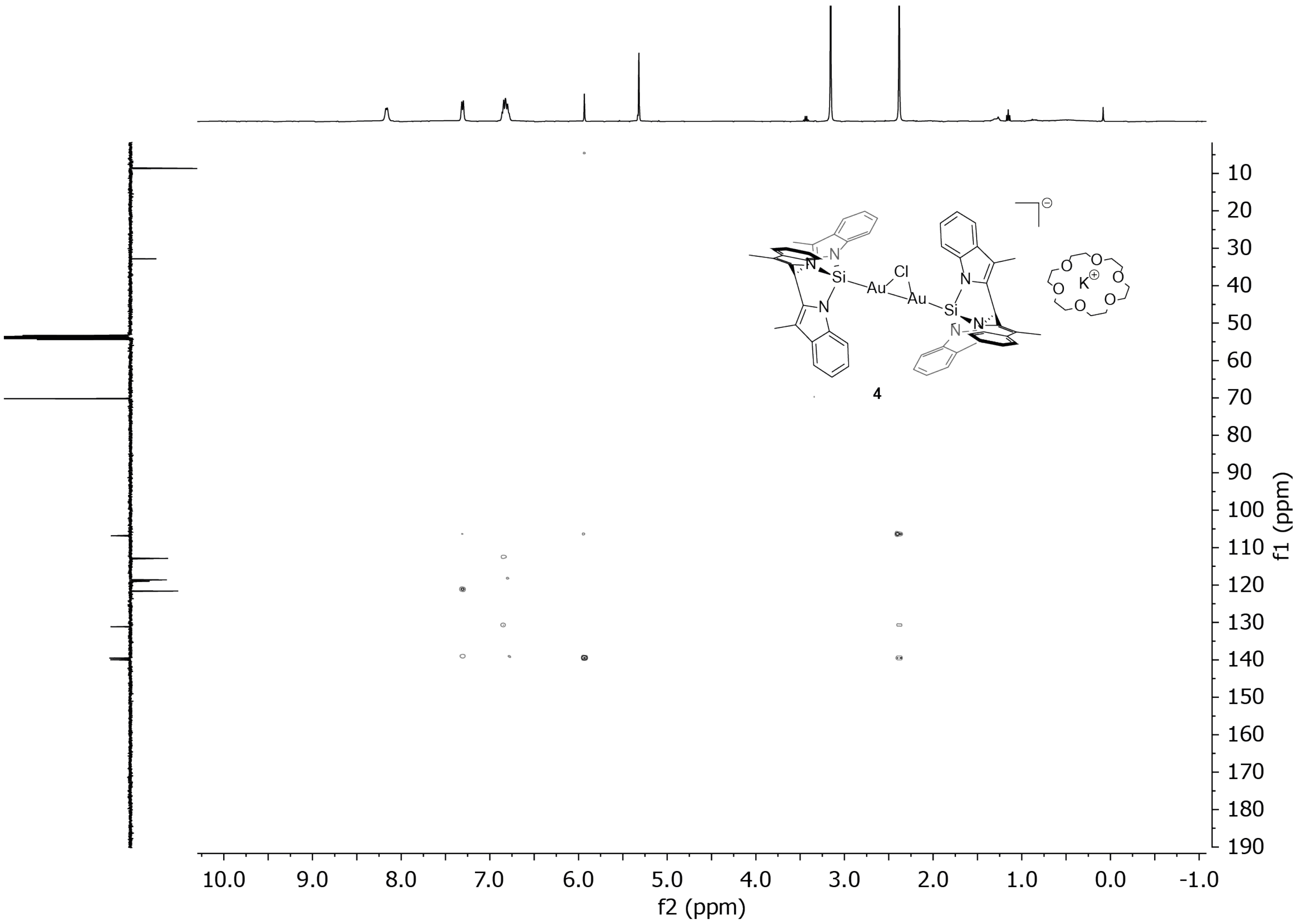

Figure S24. ^{1}H-^{13}C HMBC spectrum of compound **4** in CD_2Cl_2 at 25°C.

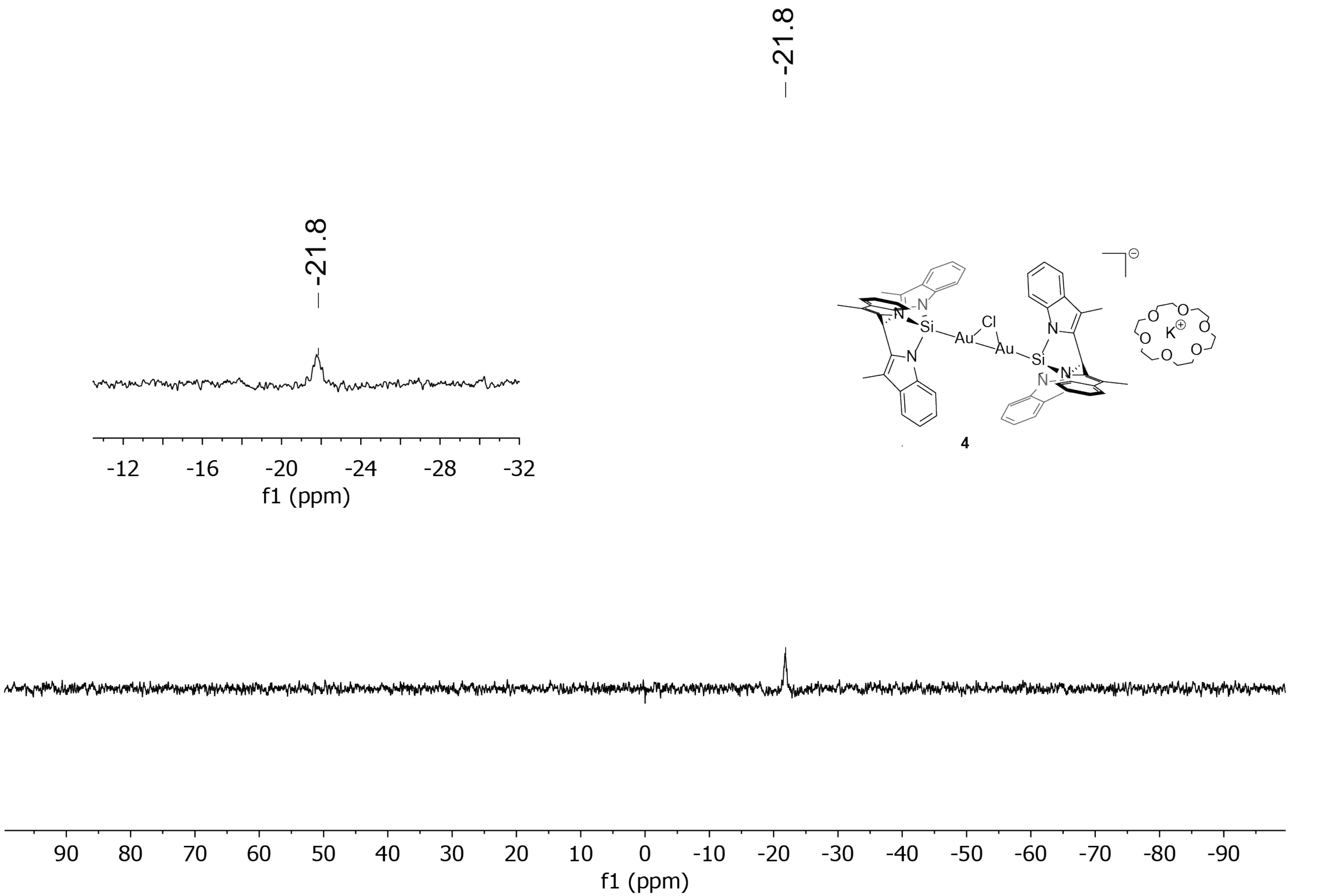

Figure S25. ^{29}Si-NMR (79 MHz) spectrum of compound **4** in CD_2Cl_2 at 25°C.

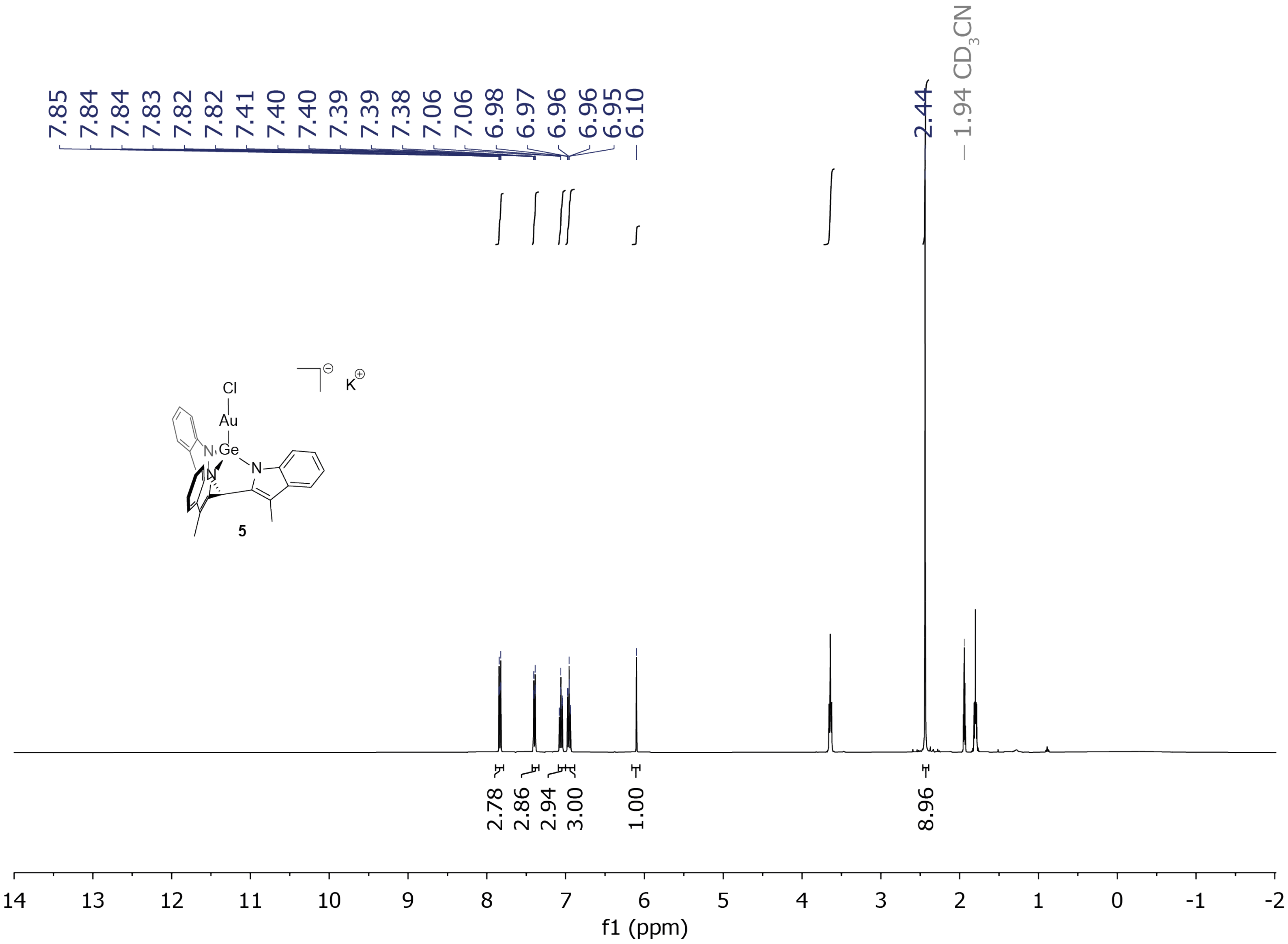

Figure S26. ^{1}H-NMR(400 MHz) spectrum of compound **5** in CD_3CN at 25°C.

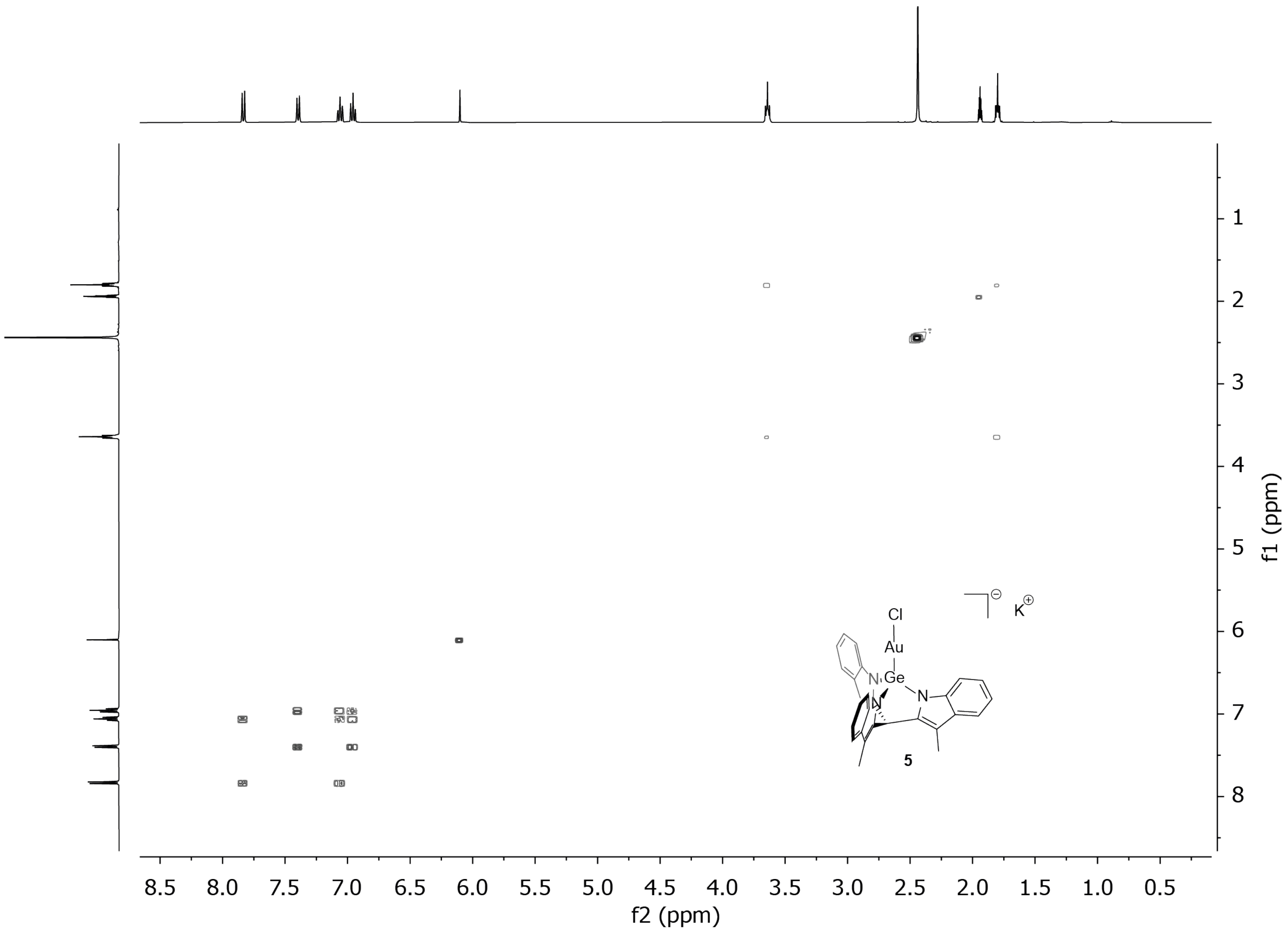

Figure S27. ^{1}H-COSY (400 MHz) spectrum of compound **5** in CD_3CN_2 at 25°C. Impurities: 3.64 and 1.80 ppm – THF.

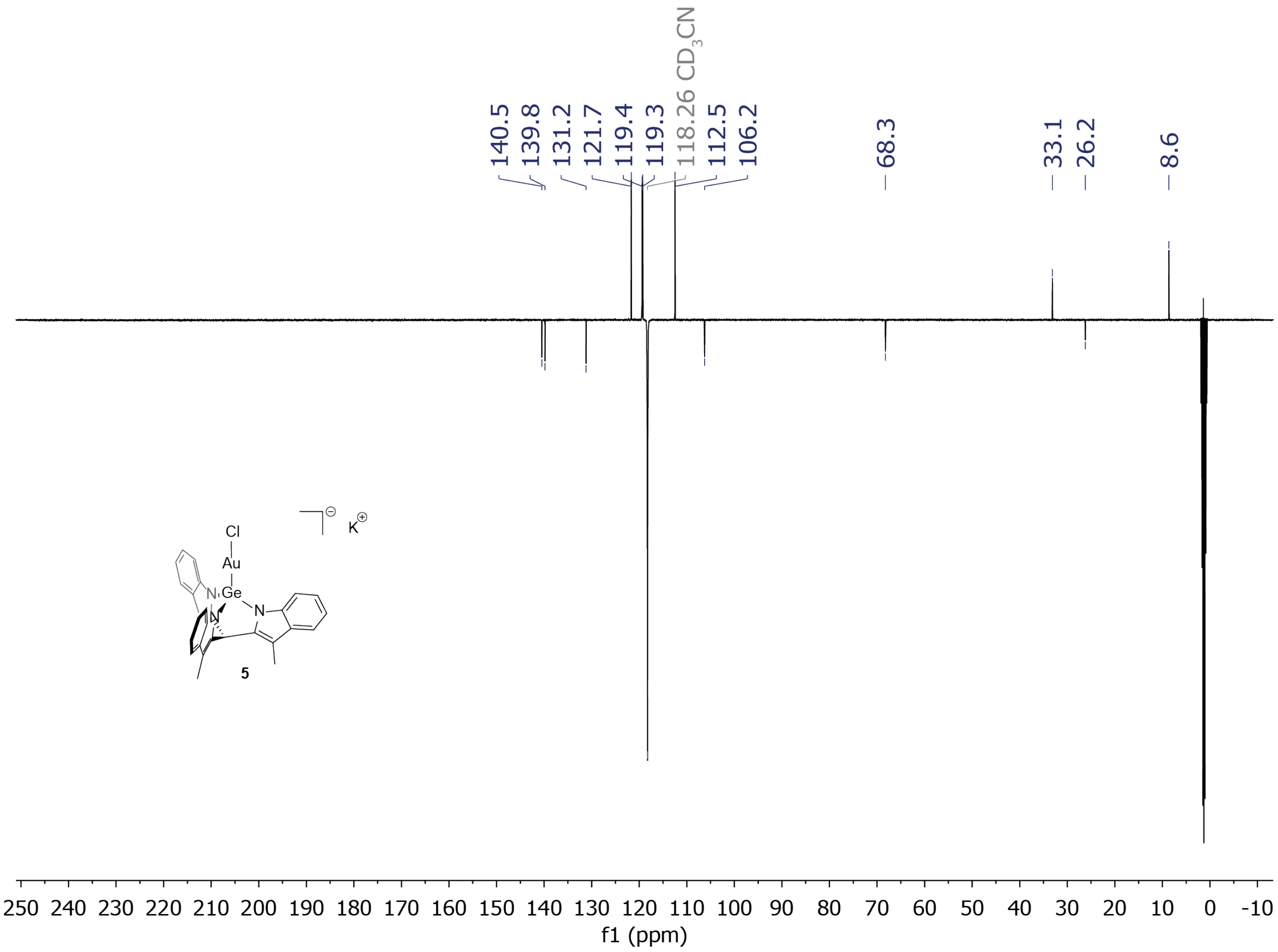

5

Figure S28. ^{13}C-NMR (101 MHz) spectrum of compound **5** in CD_3CN at 25°C. Impurities: 68.2 and 26.2 ppm – THF.

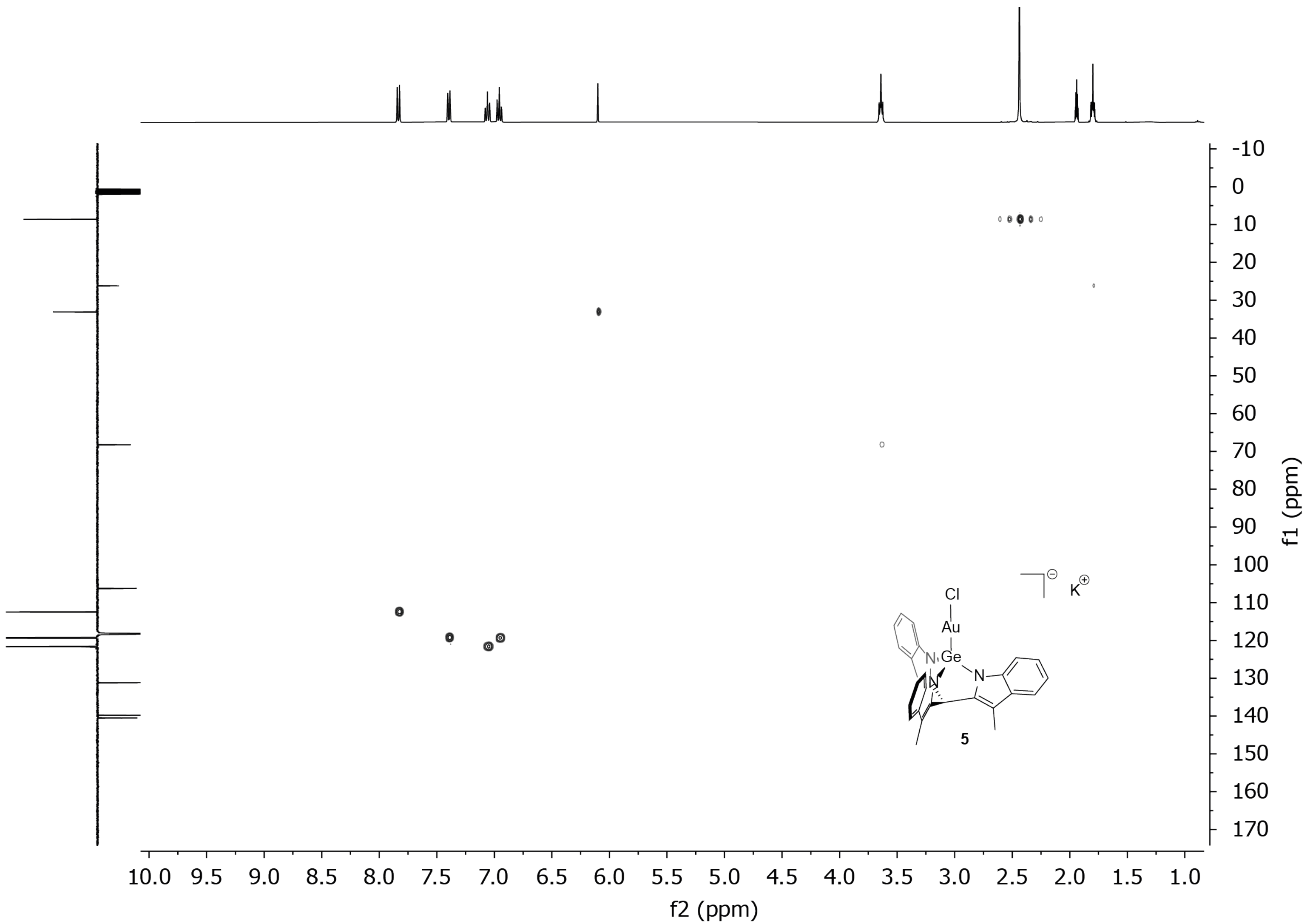

Figure S29. ^{1}H-^{13}C HSQC spectrum of compound **5** in CD_3CN at 25°C.

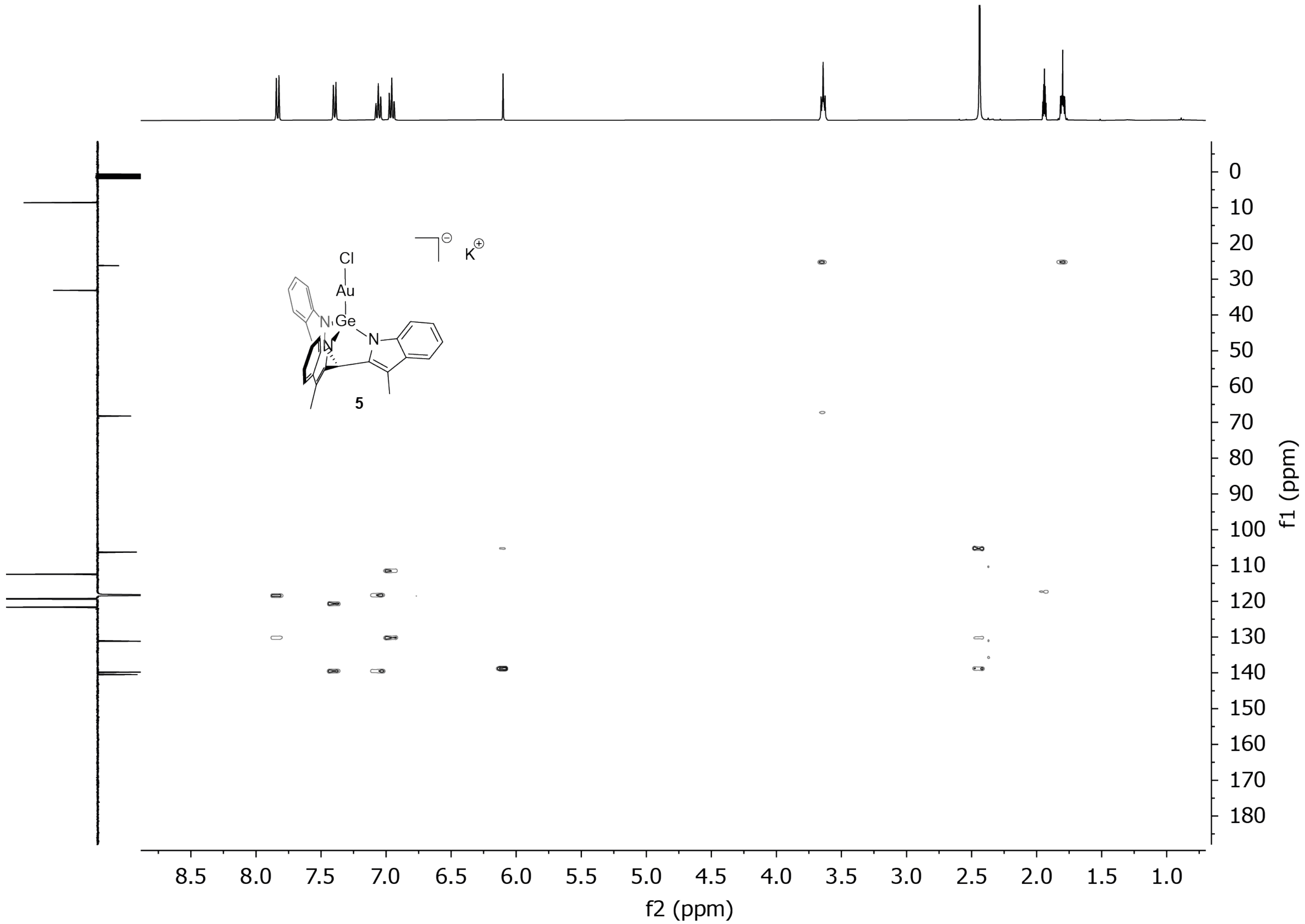

Figure S30. ^{1}H-^{13}C HMBC spectrum of compound **5** in CD_3CN at 25°C.

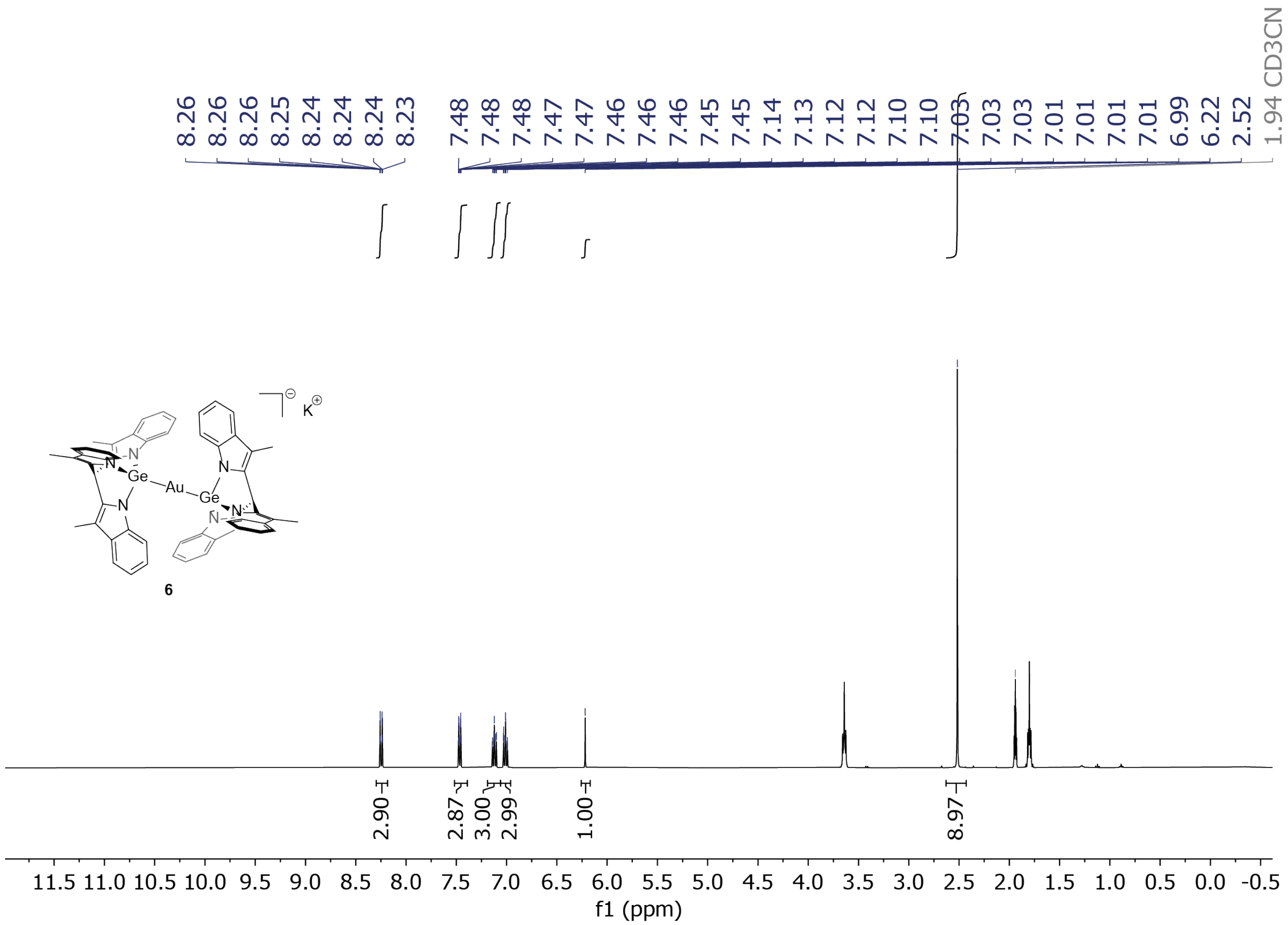

6

Figure S31. ^{1}H-NMR(400 MHz) spectrum of compound **6** in CD_3CN at 25°C. Impurities: 3.64 and 1.80 ppm – THF.

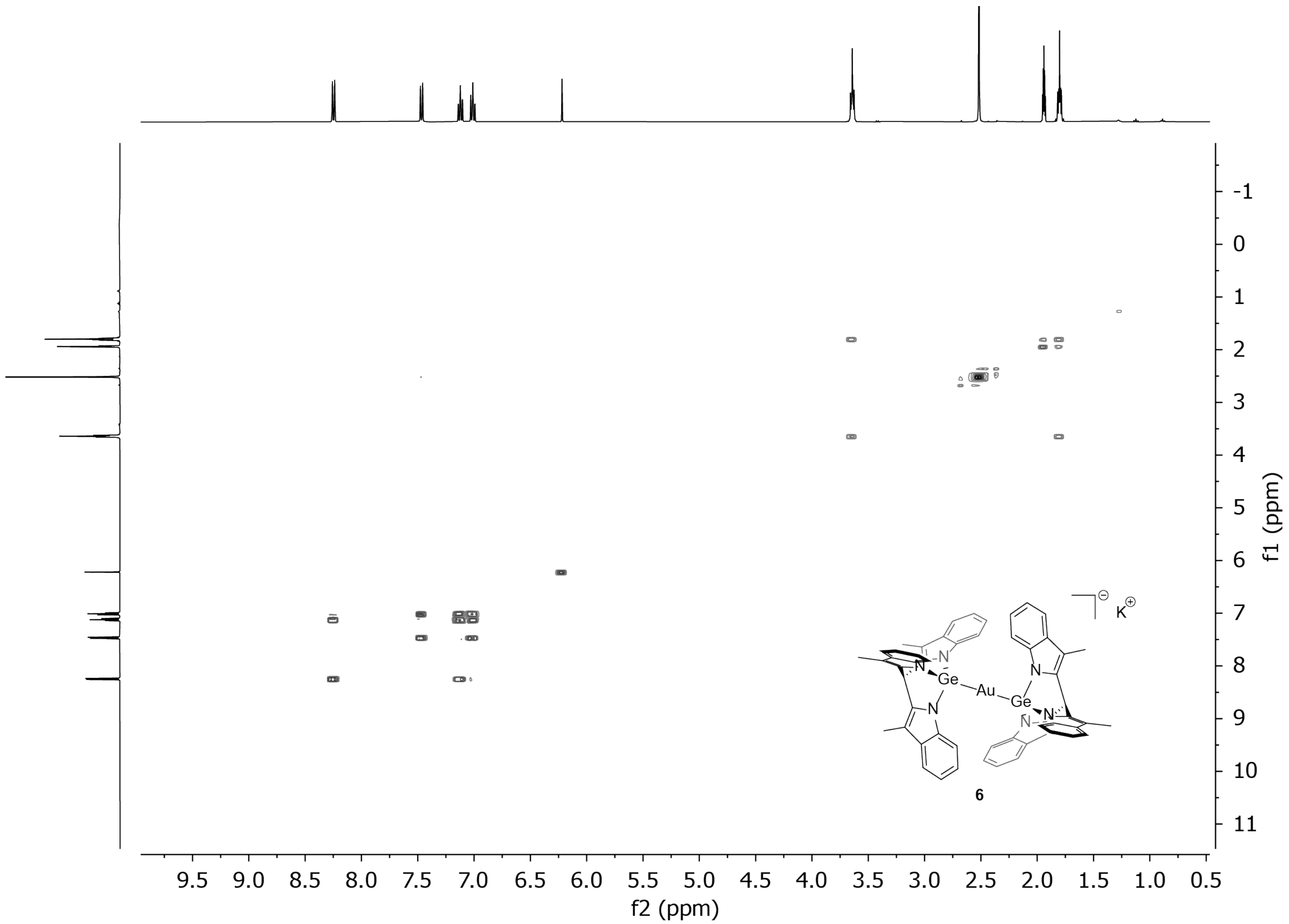

Figure S32. ^{1}H-COSY (400 MHz) spectrum of compound **6** in CD_3CN_2 at 25°C. Impurities: 3.64 and 1.80 ppm – THF.

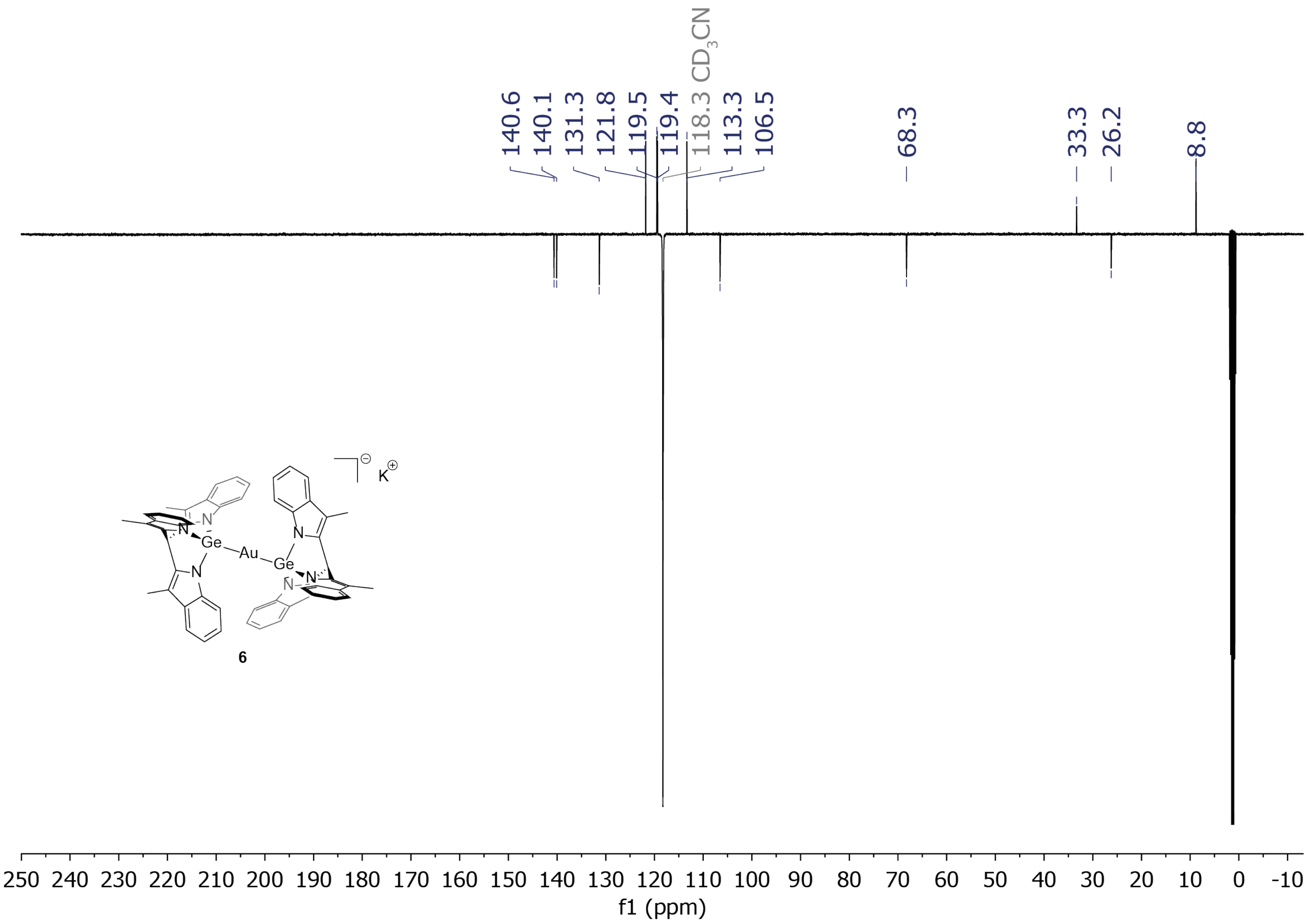

Figure S33. ^{13}C-NMR(101 MHz) spectrum of compound **6** in CD_3CN at 25°C. Impurities: 68.2 and 26.2 ppm – THF-d_8.

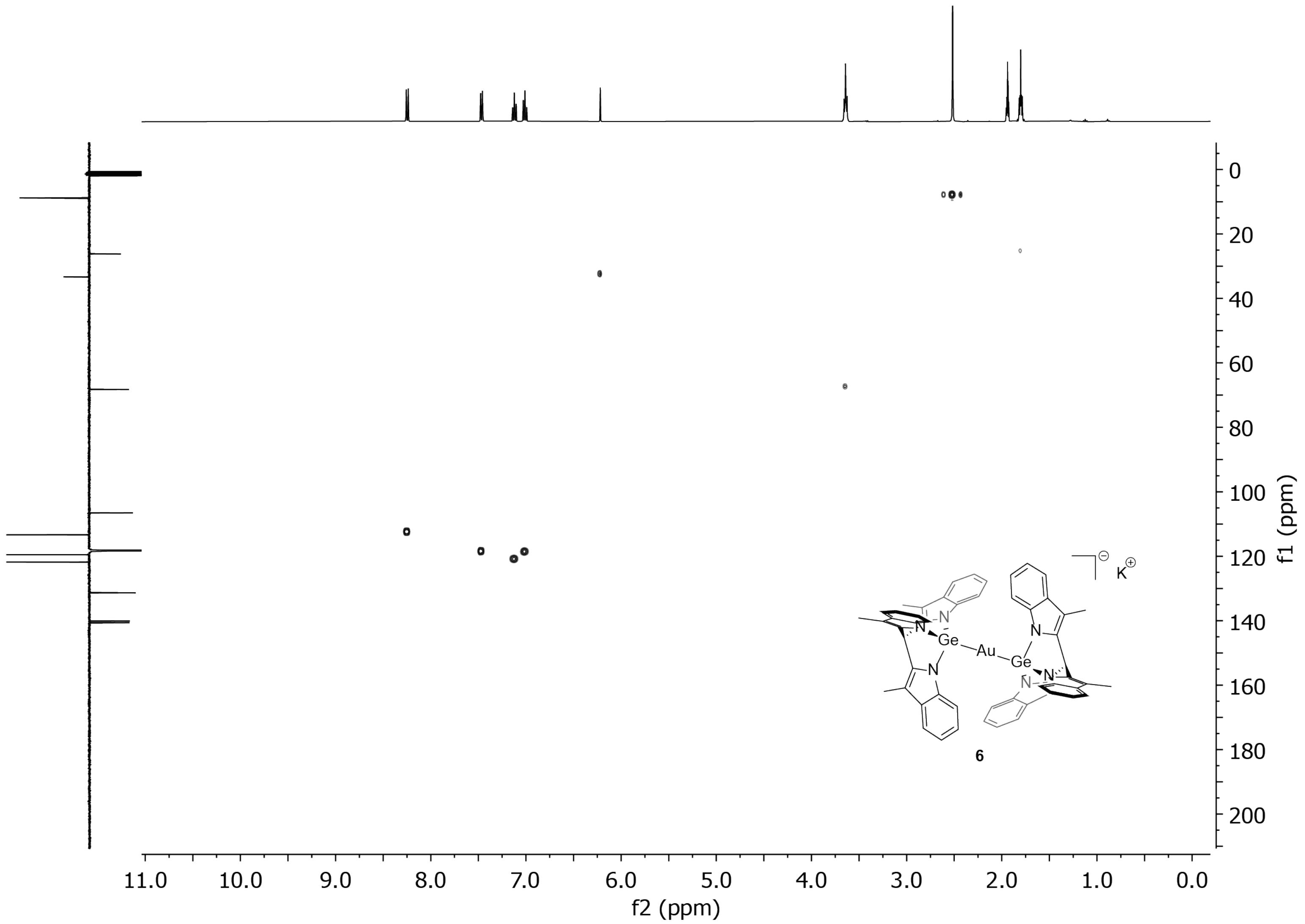

Figure S34. ^{1}H-^{13}C HSQC spectrum of compound **6** in CD_3CN at 25°C.

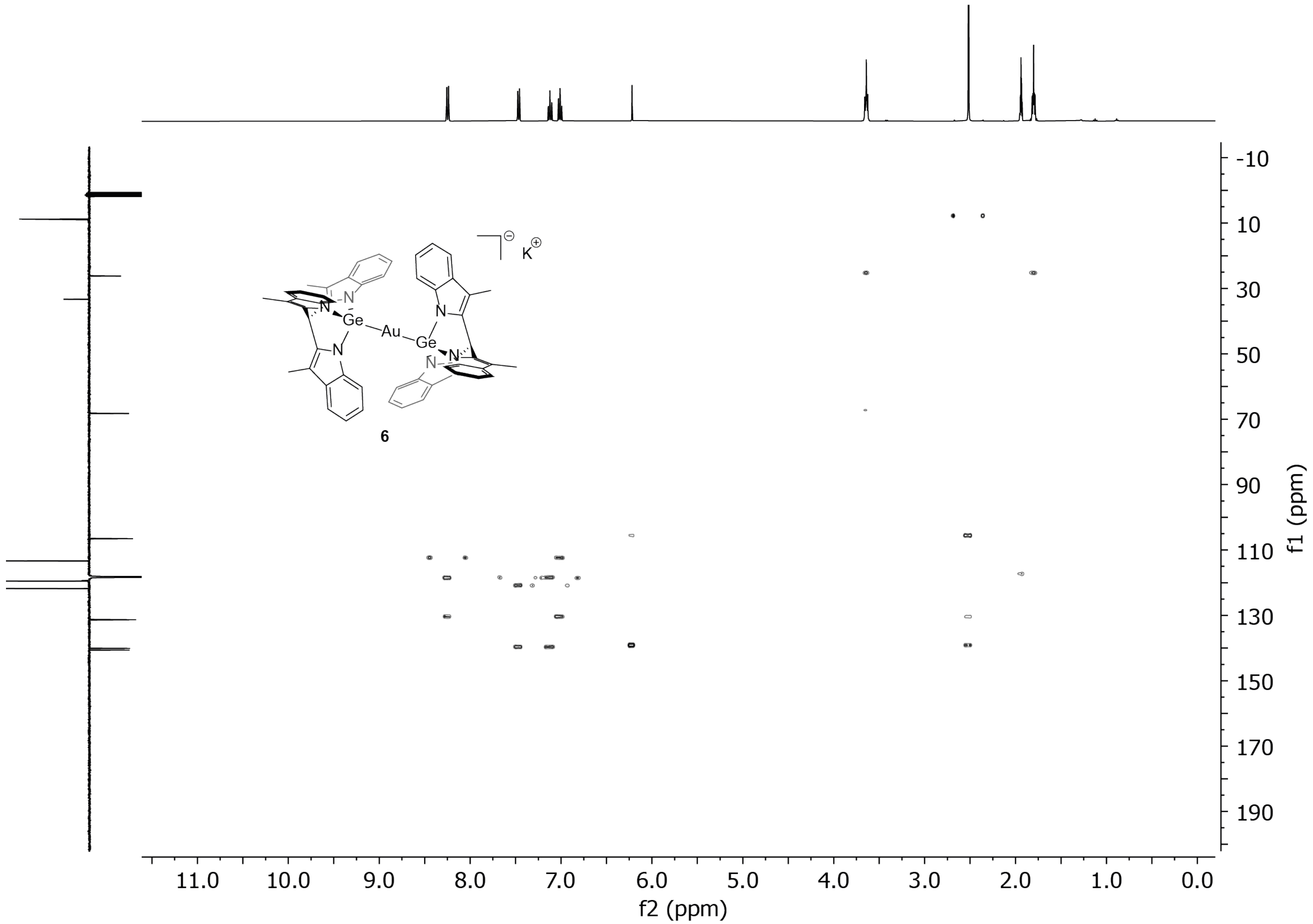

Figure S35. ^{1}H-^{13}C HMBC spectrum of compound **6** in CD_3CN at 25°C.

7. References

(1) Witteman, L.; Evers, T.; Lutz, M.; Moret, M. E. A Free Silanide from Nucleophilic Substitution at Silicon(II). *Chemistry - A European Journal* **2018**, *24* (47), 12236–12240. DOI: 10.1002/chem.201801435

(2) Witteman, L.; Van Beek, C. B.; Van Veenhuizen, O. N.; Lutz, M.; Moret, M. E. Synthesis and Complexation of a Free Germanide Bearing a Tridentate N-Heterocyclic Substituent. *Organometallics* **2019**, *38* (2), 231–239. DOI: 10.1021/acs.organomet.8b00630.

(3) Ghadwal, R. S.; Roesky, H. W.; Merkel, S.; Henn, J.; Stalke, D. Lewis Base Stabilized Dichlorosilylene. *Angewandte Chemie - International Edition* **2009**, *48* (31), 5683–5686. DOI: 10.1002/anie.200901766.

(4) Hooper, T. N.; Butts, C. P.; Green, M.; Haddow, M. F.; McGrady, J. E.; Russell, C. A. Synthesis, Structure and Reactivity of Stable Homoleptic Gold(I) Alkene Cations. *Chemistry - A European Journal* **2009**, *15* (45), 12196–12200. DOI: 10.1002/chem.200902566.

(5) Benzan Lantigua, P. A.; Lutz, M.; Moret, M. E. Polar X−H Bond (X=O, S, N) Activation at a Cage Silanide. *Angewandte Chemie - International Edition* **2024**, *63* (11). DOI: 10.1002/anie.202319899.

(6) Schreurs, A. M. M.; Xian, X.; Kroon-Batenburg, L. M. J. EVAL15: A Diffraction Data Integration Method Based on *Ab Initio* Predicted Profiles. *Journal of Applied Crystallography* **2010**, *43* (1), 70–82. DOI: 10.1107/S0021889809043234.

(7) Krause, L.; Herbst-Irmer, R.; Sheldrick, G. M.; Stalke, D. Comparison of Silver and Molybdenum Microfocus X-Ray Sources for Single-Crystal Structure Determination. *Journal of Applied Crystallography* **2015**, *48* (1), 3–10. DOI: 10.1107/S1600576714022985

(8) Sheldrick, G. M. SHELXT – Integrated Space-Group and Crystal-Structure Determination. *Acta Crystallographica Section A, Foundations and Advances* **2015**, *71* (1), 3–8. DOI: 10.1107/S2053273314026370.

(9) Sheldrick, G. M. Crystal Structure Refinement with SHELXL. *Acta Crystallographica Section C, Structural Chemistry* **2015**, *71* (1), 3–8. DOI: 10.1107/S2053229614024218.

(10) Spek, A. L. PLATON SQUEEZE: A Tool for the Calculation of the Disordered Solvent Contribution to the Calculated Structure Factors. *Acta Crystallographica Section C, Structural Chemistry* **2015**, *71* (1), 9–18. DOI: 10.1107/S2053229614024929.

(11) Spek, A. L. Structure Validation in Chemical Crystallography. *Acta Crystallographica Section D, Structural Biology* **2009**, *65* (2), 148–155. DOI: 10.1107/S090744490804362X.

(12) Parsons, S.; Flack, H. D.; Wagner, T. Use of Intensity Quotients and Differences in Absolute Structure Refinement. *Acta Crystallographica Section B, Structural Science, Crystal Engineering, and Materials* **2013**, *69* (3), 249–259. DOI: 10.1107/S2052519213010014

(13) Sevvana, M.; Ruf, M.; Uson, I.; Sheldrick, G. M.; Herbst-Irmer, R. Non-Merohedral Twinning: From Minerals to Proteins Sevvana Madhumati. *Acta Crystallographica Section D, Structural Biology* **2019**, *75* (12), 1040–1050. DOI:10.1107/S2059798319010179

(14) Frisch, M. J.; Trucks, G. W.; Schlegel, H. B.; Scuseria, G. E.; Robb, M. A.; Cheeseman, J. R.; Scalmani, G.; Barone, V.; Petersson, G. A.; Nakatsuji, H.; Li, X.; Caricato, M.; Marenich, A. V; Bloino, J.; Janesko, B. G.; Gomperts, R.; Mennucci, B.; Hratchian, H. P.; Ortiz, J. V; Izmaylov, A. F.; Sonnenberg, J. L.; Williams; Ding, F.; Lipparini, F.; Egidi, F.; Goings, J.; Peng, B.; Petrone, A.; Henderson, T.; Ranasinghe, D.; Zakrzewski, V. G.; Gao, J.; Rega, N.; Zheng, G.; Liang, W.; Hada, M.; Ehara, M.; Toyota, K.; Fukuda, R.; Hasegawa, J.; Ishida, M.; Nakajima, T.; Honda, Y.; Kitao, O.; Nakai, H.; Vreven, T.; Throssell, K.; Montgomery Jr, J. A.; Peralta, J. E.; Ogliaro, F.; Bearpark, M. J.; Heyd, J. J.; Brothers, E. N.; Kudin, K. N.; Staroverov, V. N.; Keith, T. A.; Kobayashi, R.; Normand, J.; Raghavachari, K.; Rendell, A. P.; Burant, J. C.; Iyengar, S. S.; Tomasi, J.; Cossi, M.; Millam, J. M.; Klene, M.; Adamo, C.; Cammi, R.; Ochterski, J. W.; Martin, R. L.; Morokuma, K.; Farkas, O.; Foresman, J. B.; Fox, D. J. *Gaussian 16 Rev. C.01*. Wallingford, CT **2016**.

(15) Glendening, E. D.; Landis, C. R.; Weinhold, F. NBO 6.0: Natural Bond Orbital Analysis Program. *Journal of Computational Chemistry* **2013**, *34* (16), 1429–1437. DOI: 10.1002/jcc.23266.

(16) Ehlers, A. W.; Böhme, M.; Dapprich, S.; Gobbi, A.; Höllwarth, A.; Jonas, V.; Köhler, K. F.; Stegmann, R.; Veldkamp, A.; Frenking, G. A Set of F-Polarization Functions for Pseudo-Potential Basis Sets of the Transition Metals Sc Cu, Y Ag and La Au. *Chemical Physics Letters* **1993**, *208* (1–2), 111–114. DOI: 10.1016/0009-2614(93)80086-5.

Author Contributions

Dr. M.-E. Moret initiated and supervised the project. P.A. Benzan Lantigua performed the experiments and DFT calculations. Dr. M. Lutz oversaw the acquisition, solving and interpretation of the X-ray crystal structure. P.A. Benzan Lantigua and Dr. M.-E. Moret wrote the manuscript with the contribution of Dr. M. Lutz. All authors approved the final manuscript.
